# A regulatory loop involving the cytochrome P450-soluble epoxide hydrolase axis and TGF-β signaling

**DOI:** 10.1016/j.isci.2024.110938

**Published:** 2024-09-16

**Authors:** Xiaoming Li, Sebastian Kempf, Fredy Delgado Lagos, Ürün Ukan, Rüdiger Popp, Jiong Hu, Timo Frömel, Stefan Günther, Andreas Weigert, Ingrid Fleming

**Affiliations:** 1 Goethe University, Institute for Vascular Signalling, Centre for Molecular Medicine, Frankfurt am Main, Germany; 2 Department of Embryology and Histology, School of Basic Medicine, Tongi Medical College, Huazhong University of Science and Technology, Wuhan, China; 3 Bioinformatics and Deep Sequencing Platform, Max Planck Institute for Heart and Lung Research, 61231 Bad Nauheim, Germany; 4 Goethe University, Institute of Biochemistry I, Frankfurt am Main, Germany; 5 German Center of Cardiovascular Research (DZHK), Partner site Rhein-Main, Frankfurt am Main, Germany

**Keywords:** Biochemistry, Molecular biology, Cell biology, Omics, Transcriptomics

## Abstract

Fatty acid metabolites, produced by cytochrome P450 enzymes and soluble epoxide hydrolase (sEH), regulate inflammation. Here, we report that the transforming growth factor β (TGF-β)-induced polarization of macrophages to a pro-resolving phenotype requires Alk5 and Smad2 activation to increase sEH expression and activity. Macrophages lacking sEH showed impaired repolarization, reduced phagocytosis, and maintained a pro-inflammatory gene expression profile. 11,12-Epoxyeicosatrienoic acid (EET) was one altered metabolite in sEH^−/−^ macrophages and mimicked the effect of sEH deletion on gene expression. Notably, 11,12-EET also reduced Alk5 expression, inhibiting TGF-β-induced Smad2 phosphorylation by triggering the cytosolic translocation of the E3 ligase Smurf2. These findings suggest that sEH expression is controlled by TGF-β and that sEH activity, which lowers 11,12-EET levels and promotes TGF-β signaling by metabolizing 11,12-EET to prevent Alk5 degradation. Thus, an autocrine loop between sEH/11,12-EET and TGF-β1 regulates macrophage function.

## Highlights

• Macrophages lacking soluble epoxide hydrolase fail to form a pro-resolving phenotype
• Soluble epoxide hydrolase activity determines TGF-β signaling via expression of Alk5
• 11,12-EET activates Smurf2 to initiate the degradation of Alk5

## Graphical abstract

**Figure:**
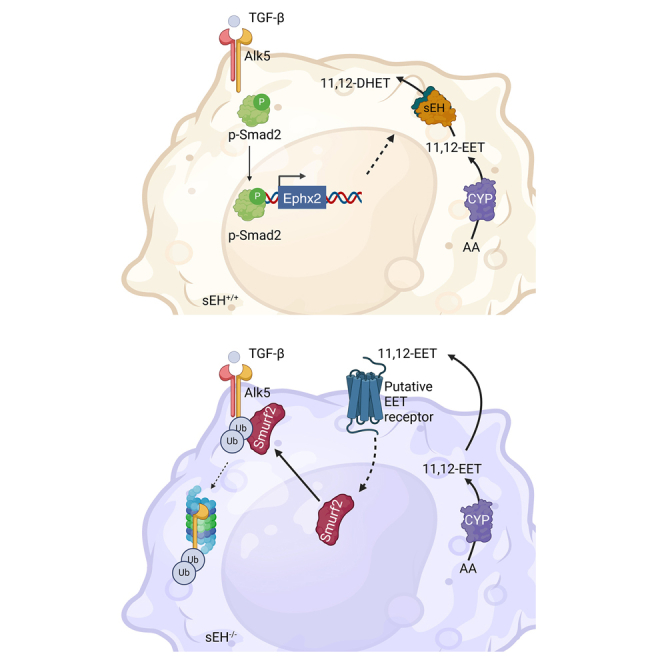

## Introduction

The soluble epoxide hydrolase (sEH; gene = *Ephx2*) is a bifunctional enzyme that acts as a lipid phosphatase and an epoxide hydrolase, metabolizing epoxides of polyunsaturated fatty acids (PUFAs) to their vicinal diols (for reviews see^1^^,^^2^). Inhibiting the sEH is generally associated with anti-inflammatory effects attributed to the accumulation of its substrates and/or the absence of specific cytotoxic diols. For example, the accumulation of the epoxides of arachidonic acid, i.e., epoxyeicosatrienoic acids (EETs), has been linked with beneficial effects in the lung,^3^ kidney, and cardiovascular system.^2^ Indeed, deletion of the sEH attenuates lipopolysaccharide (LPS)-induced pulmonary inflammation,^4^^,^^5^ and sEH deletion or inhibition attenuates atherosclerosis.^6^^,^^7^^,^^8^^,^^9^^,^^10^ Similar protective effects have also been described in the kidney.^11^^,^^12^ On the other hand, sEH inhibition can prevent the generation and deleterious actions of high concentrations of the linoleic acid diol 12,13-dihydroxyoctadecenoic acid (12,13-DiHOME), which seems to play crucial role in adult respiratory distress syndrome associated with burn injury^13^^,^^14^ and COVID-19.^15^^,^^16^^,^^17^ The sEH also generates the docosahexaenoic-acid-derived diol, 19,20-dihydroxydocosapentaenoic acid, which has been causally implicated in non-proliferative diabetic retinopathy^18^ and the retinal vasculopathy associated with polycystic kidney disease.^19^

The inflammatory reaction is characterized by distinct phases, each associated with a specific lipid profile. Although significant attention has been given to the roles of cyclooxygenase and lipoxygenase products, as well as sphingolipids and specialized pro-resolving mediators, less focus has been placed on the mediators generated by the sequential actions of cytochrome P450 (CYP) enzymes and the sEH. The role of sEH in the innate immune system is not well understood, but altering its activity is known to affect neutrophil infiltration.^5^^,^^20^^,^^21^ Monocytes and macrophages also express the enzyme, and its levels remain stable during the differentiation of peripheral blood monocytes into macrophages.^22^ sEH inhibition has been shown to prevent the classical activation, or M1 polarization, of macrophages^23^^,^^24^^,^^25^^,^^26^^,^^27^ and inhibit LPS-induced cytokine release.^28^^,^^29^ Similar effects have been attributed to the EETs that accumulate following sEH inhibition.^25^ The result is a bias in the differentiation of macrophages toward an anti-inflammatory (alternatively activated or M2) phenotype.^24^^,^^30^ However, many of the findings reported *in vivo* can also reflect the consequences of sEH inhibition or deletion on the proliferation of stem cells and the mobilization of bone-marrow-derived cells^31^ rather than direct effects on cell function. The aim of this study was to more thoroughly investigate the role of the sEH in macrophage polarization and function, focusing more closely on the pathways regulating sEH expression and the impact of sEH deletion on the process of resolution.

## Results

### Effect of altered sEH expression on inflammation and its resolution in a mouse model of peritonitis

**Figure 1:**
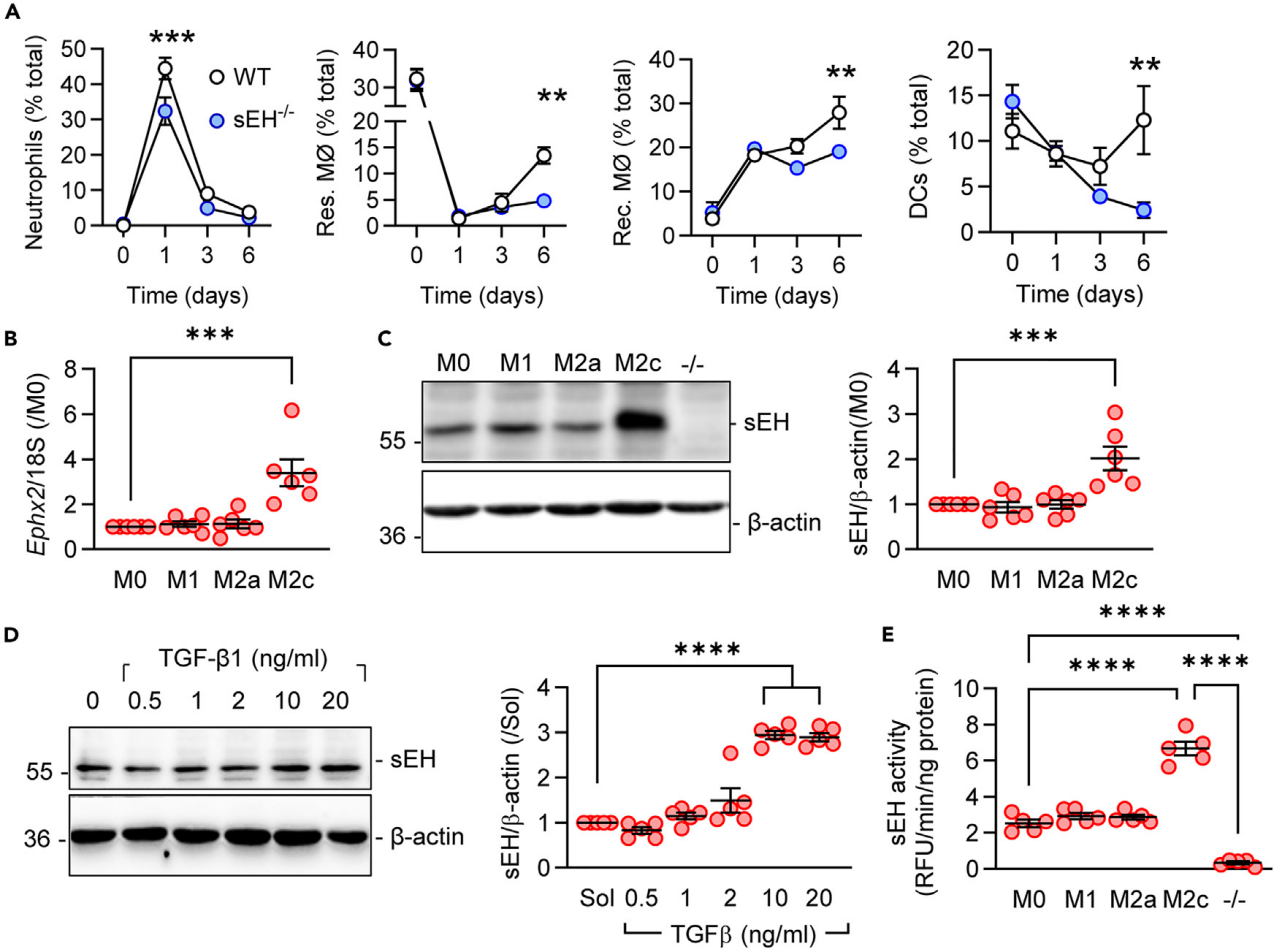
Consequence of impaired sEH activity on the resolution of inflammation and sEH expression in macrophages (A) Cells present in peritoneal fluid from wild-type and sEH^−/−^ mice up to 6 days after a single intraperitoneal injection of zymosan A (10 mg/kg). Neutrophils, Res. MØ, resident macrophages; Rec. MØ, recruited macrophages; DCs, dendritic cells; n = 5–6 animals per group. (B and C) *Ephx2* RNA (B) and sEH protein (C) in murine bone-marrow-derived macrophages under basal conditions M0 (Sol) and after polarization to the M1, M2a, or M2c states; *n* = 6 mice per group. M2c macrophages from sEH^−/−^ mice were included as a negative control. (D) Concentration-dependent effects of TGF-β1 (48 h) on sEH expression in M1-polarized murine macrophages; *n* = 5 mice per group. (E) sEH activity in polarized macrophages; *n* = 5 independent experiments. M2c-polarized macrophages from sEH^−/−^ mice were included as a negative control. Error bars = SEM, (A) Two-way ANOVA followed by Sidak’s multiple comparisons test; (B–E) one-way ANOVA and Tukey’s multiple comparisons test. ∗∗*p* < 0.01, ∗∗∗*p* < 0.001, ∗∗∗∗*p* < 0.0001.

To determine the *in vivo* relevance of sEH deficiency on the resolution of inflammation, 8- to12-week-old wild-type and sEH^−/−^ mice were challenged with zymosan to induce peritonitis, and the cellular constituents of the peritoneal lavage fluid were monitored over 6 days. Peritonitis resulted in neutrophil recruitment on day one, which was significantly attenuated in sEH^−/−^ mice (Figure 1A). On day 3, more CD8^+^ T cells but fewer T regulatory cells and natural killer cells were recovered from sEH^−/−^ mice (Figures S1). At later time points, lavage from sEH^−/−^ mice contained fewer recruited macrophages, dendritic cells, and conventional dendritic cells and on day 6 also significantly fewer resident macrophages. These observations revealed a significant effect of sEH in promoting both the initiation and resolution of inflammation.

### Impact of macrophage polarization on sEH expression

The sEH is expressed in bone marrow progenitor cells and its deletion attenuates the mobilization of myeloid cells from the bone marrow,^31^ meaning that the effects observed *in vivo* were likely to reflect a combination of sEH-dependent actions on myeloid mobilization and differentiation. Therefore, we concentrated on the phenotype of murine bone-marrow-derived macrophages. *Ephx2*/sEH was expressed at comparable levels by naive (M0) macrophages as well as in classically activated M1 and alternatively activated or M2a-polarized macrophages (Figures 1B and 1C). However, its levels were significantly higher in macrophages repolarized from M1 to a pro-resolution M2c phenotype with transforming growth factor β1 (TGF-β1). The latter phenomenon was concentration dependent (Figure 1E) and accompanied by a marked increase in sEH activity (Figure 1D).

**Figure 2:**
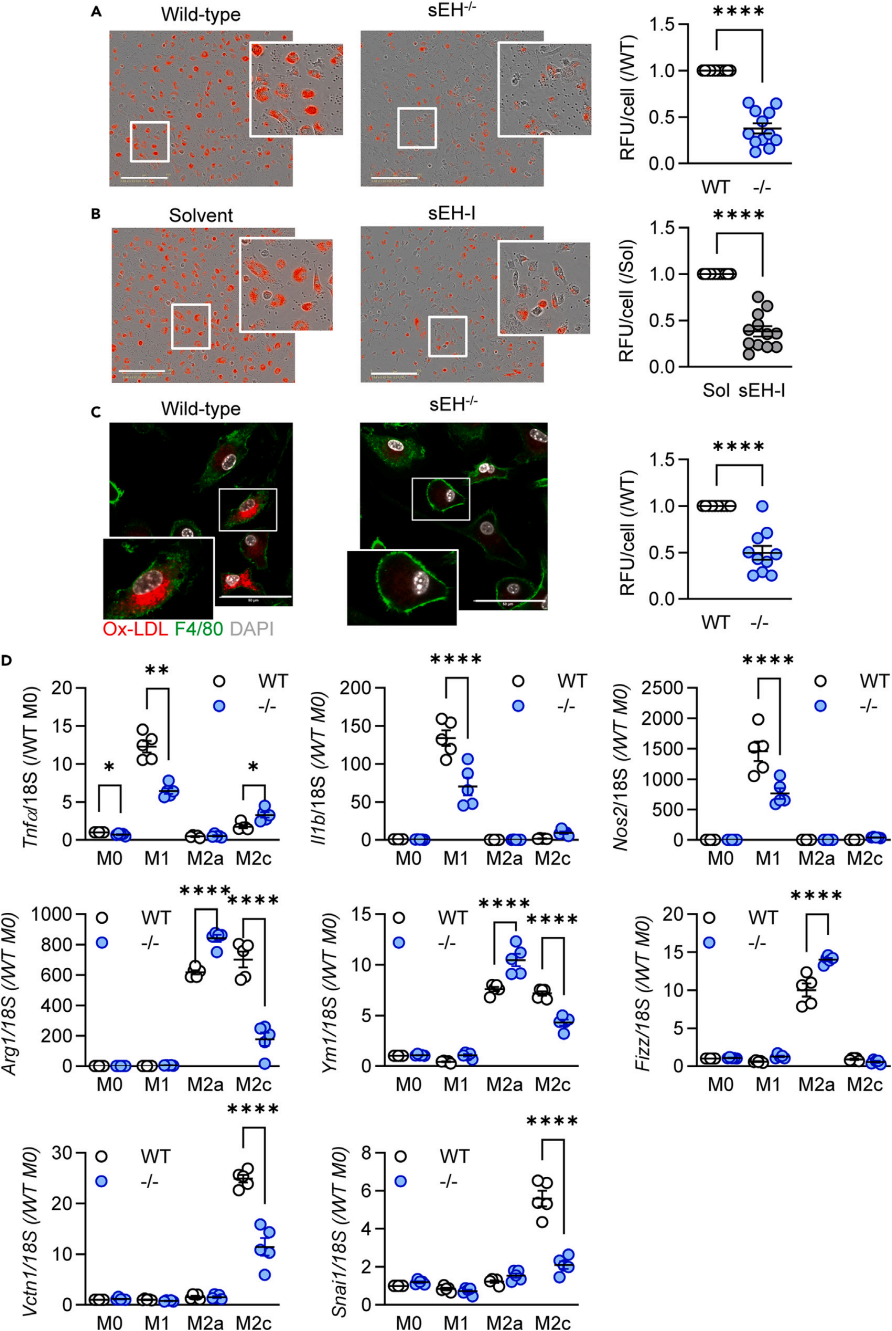
Consequence of impaired sEH activity on macrophage phagocytosis and polarization (A and B) pHrodo Red Zymosan uptake by M2c-polarized macrophages from (A) wild-type (WT) and sEH^−/−^ (−/−) mice or (B) from WT M2c macrophages treated with solvent or an sEH inhibitor (sEH-I, *t*-AUCB, 5 μmol/L); scale bar = 200 μm; *n* = 12 mice per group. (C) Oxidized LDL (ox-LDL) uptake by M2c-polarized macrophages from WT and sEH^−/−^ mice; scale bar = 50 μm; *n* = 10 mice per group. (D) Expression marker genes in bone-marrow-derived macrophages from wild-type (WT) and sEH^−/−^ (−/−) mice under basal conditions (M0) and following M1, M2a, and M2c polarization; n = 5–6 mice per group. Error bars = SEM, (A–C) Student’s t test; (D) two-way ANOVA followed by Sidak’s multiple comparisons test. ∗*p* < 0.05, ∗∗*p* < 0.01, ∗∗∗∗*p* < 0.0001.

A major role of macrophages during the resolution of inflammation is the efferocytosis of apoptotic neutrophils and phagocytosis of other cellular debris.^32^ Consistent with the consequences of sEH deletion on the resolution of inflammation, the phagocytosis of zymosan was clearly attenuated in M2c macrophages from sEH^−/−^ mice (Figure 2A). A similar effect on zymosan phagocytosis was also observed in macrophages from wild-type mice treated with the sEH inhibitor, *t*-AUCB (Figure 2B). The effect was not restricted to zymosan, as the uptake of ox-LDL was also greater in macrophages from wild-type versus sEH^−/−^ mice (Figure 2C). Thus, not only did the lack of sEH decrease macrophage recruitment, those cells that were recruited displayed decreased phagocytosis and thus would be expected to be less efficient in clearing cell debris and to delay the resolution of inflammation.

Male mice are known to express higher levels of sEH than female mice,^33^^,^^34^ a finding that was also apparent in bone-marrow-derived macrophages (Figure S2A). Indeed, although TGF-β increased sEH expression in cells from male mice, the response was less pronounced than in macrophages from females. A similar relationship was detected in peripheral-blood-monocyte-derived macrophages from human subjects (Figures S2B and S2C). For this reason, further experiments were performed using blood/bone marrow from female subjects/mice.

The next step was to determine whether or not the sEH was required for macrophage polarization. Consistent with previous reports,^23^^,^^24^^,^^25^^,^^26^^,^^27^ a panel of polarization markers revealed that the deletion of sEH attenuated macrophage M1 polarization, i.e., the LPS and interferon (IFN)-γ-induced increase in *Tnfa*, *Il1b*, and *Nos2* expression (Figure 2D). On the other hand, the expression of more classical M2a markers, i.e., *Arg1*, *Ym-1*, and *Fizz1*, was increased in sEH^−/−^ macrophages polarized with IL-4. The repolarization of M1 macrophages to M2c using TGF-β1 was associated with a significant decrease in *Arg1*, *Vtcn1*, and *Snai1* expression. Taken together, our data indicate that the absence of sEH significantly attenuated both M1 polarization and M2c repolarization and was consistent with the observations made in the peritonitis model.

### TGF-β1 induces sEH expression via Alk5 and Smad2

**Figure 3:**
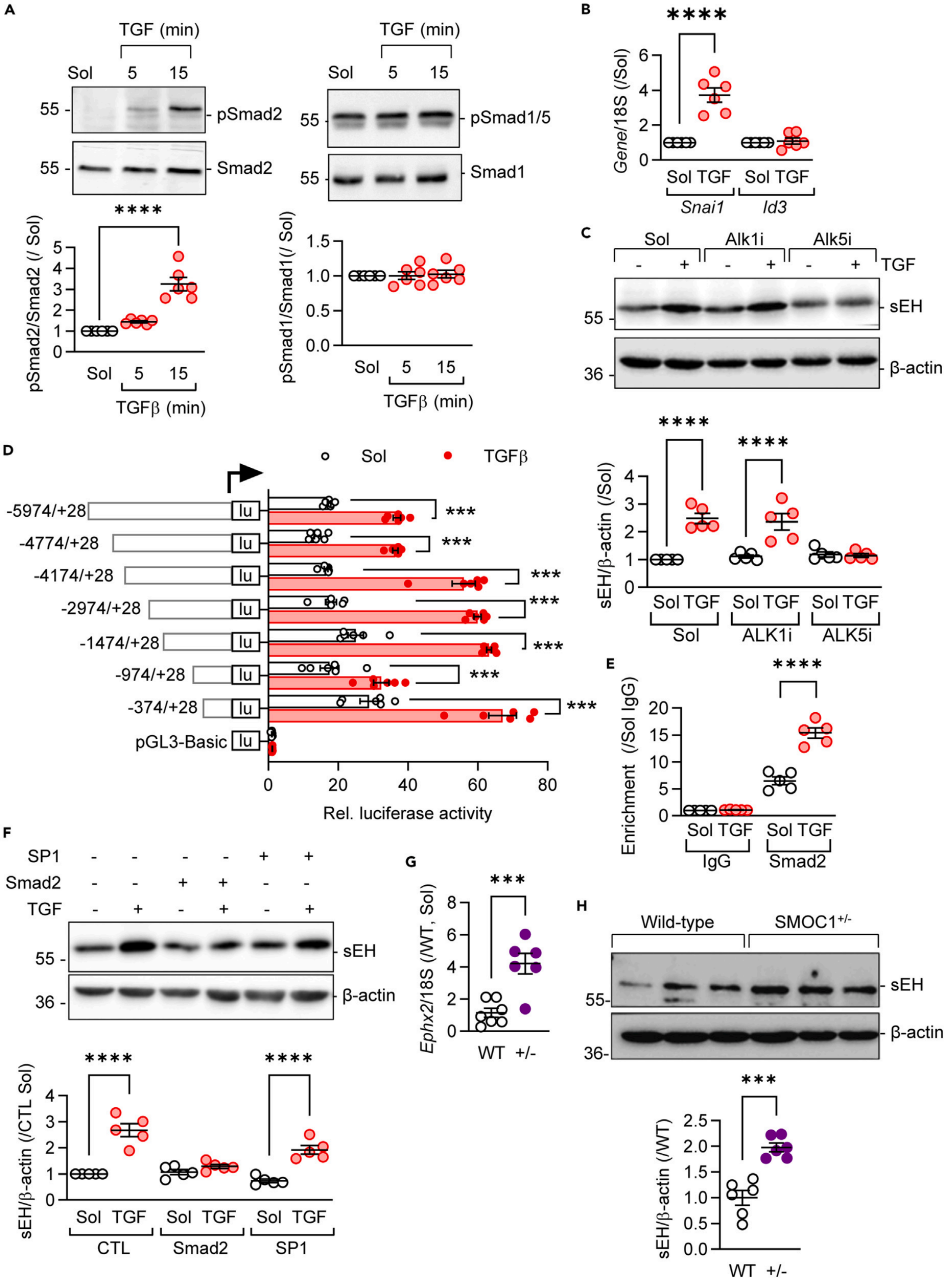
Role of ALK5-Smad2 pathway in the regulation of sEH expression by TGF-β1 (A) Effect of TGF-β1 (10 ng/mL) on the phosphorylation of SMAD2 (p-SMAD2) and SMAD1/5 (p-SMAD1/5) in M1-polarized murine macrophages; n = 5–6 mice per group. (B) Expression of *Snail* and *Id3* in M2c-polarized macrophages; n = 5–6 mice per group. (C) Consequence of ALK1 inhibition (ALK1i) with LDN193189 (1 nmol/L) and ALK5 inhibition (ALK5i) with SD208 (500 nmol/L) on the TGF-β1-induced increase in sEH expression in murine macrophages; n = 5–6 mice per group. (D) Effect of TGF-β1 (10 ng/mL, 24 h) on sEH promoter activity in HEK cells; *n* = 6 independent experiments. (E) ChIP-qPCR analysis of SMAD2 binding to human sEH promoter in M1-polarized human macrophages stimulated with TGF-β1 (10 ng/mL, 24 h); n = 5–6 donors. (F) Effect of decoy oligonucleotides directed against SMAD2 and SP1 on the TGF-β1-induced increase of sEH expression in murine M1 macrophages; n = 5–6 mice per group. (G and H) Expression of *Ephx2* (g) and sEH (h) in M2c-polarized macrophages from wild-type (WT) and SMOC1^+/−^ (+/−) littermates; *n* = 6 mice per group. Error bars = SEM, (A) One-way ANOVA followed by Turkey’s multiple comparisons test; (B, C, E, G, and H) Student’s t test; (D and F) two-way ANOVA followed by Sidak’s multiple comparisons test. ∗*p* < 0.05, ∗∗*p* < 0.01, ∗∗∗*p* < 0.001, ∗∗∗∗*p* < 0.0001.

Given the impact of TGF-β on *Ephx2*/sEH levels, we assessed the mechanism of induction. The addition of TGF-β1 to M1-polarized murine macrophages resulted in the phosphorylation of Smad2 with no apparent change in the phosphorylation of Smad1/5 (Figure 3A). Fitting with an effect mediated by the TGF-β type I receptor, activin receptor-like kinase (Alk) 5, TGF-β1 increased the expression of the Smad2/3 target *Snai1*, whereas the expression of the Alk1-Smad1/5 target *Id3* was unaffected (Figure 3B). Moreover, the increase in sEH elicited by TGF-β1 was no longer observed when experiments were performed in the presence of the Alk5 antagonist SD208 (Figure 3C). To determine the molecular mechanism(s) underlying the TGF-β1-mediated induction of sEH, a series of luciferase-coupled human *Ephx2* promoter reporter constructs were studied in HEK-293 cells. Interestingly, the expression of all of the constructs was increased by TGF-β1, and the effect was most marked in the shortest construct (−374 to +28 bp) (Figure 3D), which contained binding sites for SP1 and Smad (Figure S3). Chromatin immunoprecipitation experiments using human macrophages demonstrated that TGF-β1 increased the binding of Smad2 to the *Ephx2* promoter (Figure 3E). Fitting with this, treating murine macrophages with decoy oligonucleotides directed against Smad2 abolished the ability of TGF-β1 to increase sEH expression (Figure 3F). Decoy oligonucleotides against SP1, which was previously reported to regulate sEH expression,^23^^,^^35^ were without effect. To assess the link with TGF-β signaling more closely, sEH expression was assessed in macrophages isolated from mice lacking the endogenous Alk5 antagonist, secreted modular calcium-binding protein 1 (SMOC1).^36^ Indeed, *Ephx2*/sEH levels were elevated in M2c macrophages from SMOC1^+/−^ mice (Figures 3G and 2H) that lack detectable levels of the SMOC1 protein.^36^ These results indicated that TGF-β1 activates Alk5 to phosphorylate Smad2 and increases *Ephx2*/sEH expression.

### sEH-dependent changes in M2c macrophage gene expression

**Figure 4:**
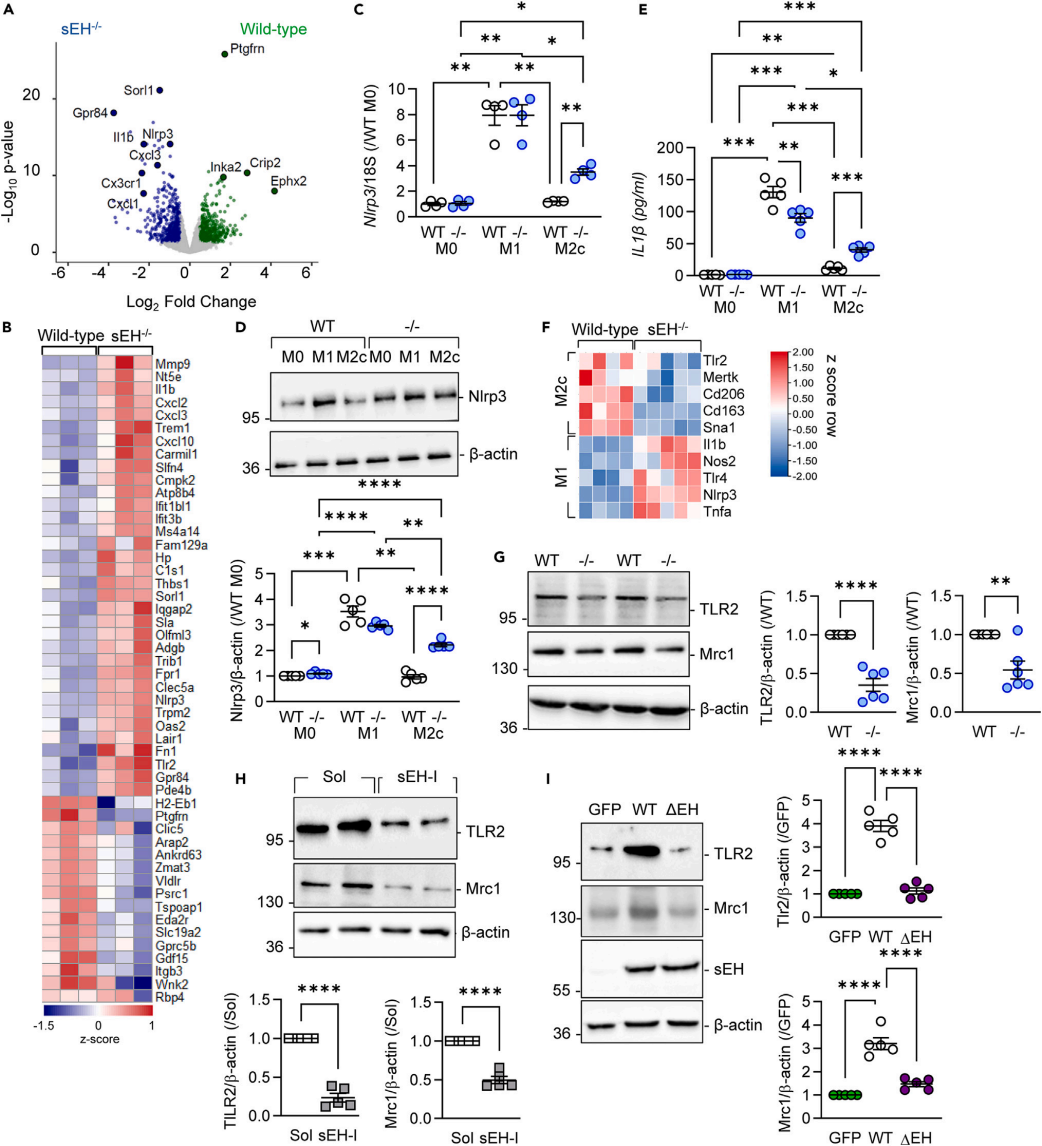
RNA-seq analysis and validation (A) Volcano plot showing differential gene expression in M2c macrophages from wild-type and sEH^−/−^ mice; *n* = 3 mice per group. Significantly regulated genes are indicated in blue (higher in sEH^−/−^) and green (higher in wild-type). (B) Heatmap showing the top 50 differentially expressed genes (Z scores) as in (A). (C–E) Bone-marrow-derived monocytes from WT and sEH^−/−^ mice were differentiated to M0, M1, or M2c macrophages. *Nlrp3* gene expression (C), Nlrp3 protein levels (D), and IL-1β levels in the cell supernatant (E); n = 4–5 mice per group. (F) Heatmap showing most differentially regulated inflammation- and resolution-related transcripts in cells from peritoneal fluid 6 days after zymosan administration; n = 4–5 mice per group. (G) Expression of Tlr2 and Mrc1 in M2c-polarized macrophages from wild-type (WT) and sEH^−/−^ (−/−) mice; n = 5–6 mice per group. (H) Tlr2 and Mrc1 in macrophages from wild-type mice polarized to M2c in the presence of solvent (Sol) or an sEH inhibitor (sEH-I); n = 5–6 mice per group. (I) Tlr2 and Mrc1 expression in M2c-polarized macrophages from sEH^−/−^ mice overexpressing either GFP, the wild-type sEH (WT), or a catalytically inactive sEH mutant (ΔEH); n = 5–6 mice per group. Error bars = SEM, (C–E) Two-way ANOVA followed by Tukey’s multiple comparisons test; (G and H) Student’s t test; (I) one-way ANOVA followed by Turkey’s multiple comparisons test. ∗∗*p* < 0.01, ∗∗∗*p* < 0.001, ∗∗∗∗*p* < 0.0001.

Bulk RNA sequencing was performed to identify transcripts differentially regulated in M2c-polarized macrophages from wild-type and sEH^−/−^ mice (Figures 4A and 4B, GEO: GSE273900). Hallmark gene set analysis revealed marked changes in processes related to inflammation with weaker effects on epithelial to mesenchymal transition as well as responses to estrogen and androgens (Figure S4). Genes significantly downregulated in sEH-deficient macrophages encoded cysteine-rich intestinal protein 2 (Crip2), which acts as a repressor of nuclear factor κB (NF-κB)-mediated cytokine transcription,^37^ prostaglandin F2 receptor negative regulator (*Ptgfrn*), and Inka box actin regulator 2 (*Inka2*), a kinase interacting protein that affects actin dynamics.^38^^,^^39^ Among the pro-inflammatory genes whose expression was increased in M2c macrophages from sEH^−/−^ mice were *IL-1β* and *Nlrp3* (see Figure 4A). NLRP3 is a component of the inflammasome that drives the activation of caspase-1, leading to the production of IL-1β (for review see^40^) and was reported to be downregulated in LPS-stimulated macrophages pre-treated with a dual cyclooxygenase and sEH inhibitor.^41^ Although *Nlrp3* expression was comparable in M1-polarized macrophages from wild-type and sEH^−/−^ mice, the M2c re-polarization-induced decrease in its expression was impaired in the absence of the sEH (Figures 4C and 4D). Levels of IL-1β were consistent with the pattern of changes in Nlrp3 (Figure 4E). Thus, the overall profile of sEH-deficient M2c-polarized macrophages was skewed to a more inflammatory phenotype.

Given that sEH deficiency altered phagocytosis, the expression of scavenger receptors and other M1 and M2 markers was also assessed in monocytes recovered from peritoneal fluid 6 days after zymosan administration. The expression of Toll-like receptor 2 (*Tlr2*), which plays an important role in phagocytosis,^42^ was decreased in cells recovered from the peritoneal fluid 6 days after zymosan administration (Figure 4F). This contrasted with the increase in *Tlr2* detected in *in vitro* differentiated M2c macrophages. Levels of *Cd163*, a scavenger receptor for haptoglobin-hemoglobin complexes mostly expressed by macrophages,^43^ and *Cd206*/*Mrc1* were also decreased while *Tlr4* expression increased. Genes encoding other phagocytosis-associated receptors, e.g., *Cd36* and *Clec7a*, were unaffected by sEH deletion (Figure S5). The decrease in TLR2 and Mrc1 was confirmed at the protein level (Figure 4G). A causative link between sEH and the expression of *Tlr2* and *Mrc1* levels was demonstrated inasmuch as both were consistently lower in wild-type M2c-polarized macrophages treated with an sEH inhibitor (Figure 4H). Moreover, *Tlr2* and *Mrc1* were increased in cells overexpressing the wild-type sEH but not a catalytically inactive sEH mutant (Figure 4I).

### PUFA metabolism in M2c repolarized macrophages

**Figure 5:**
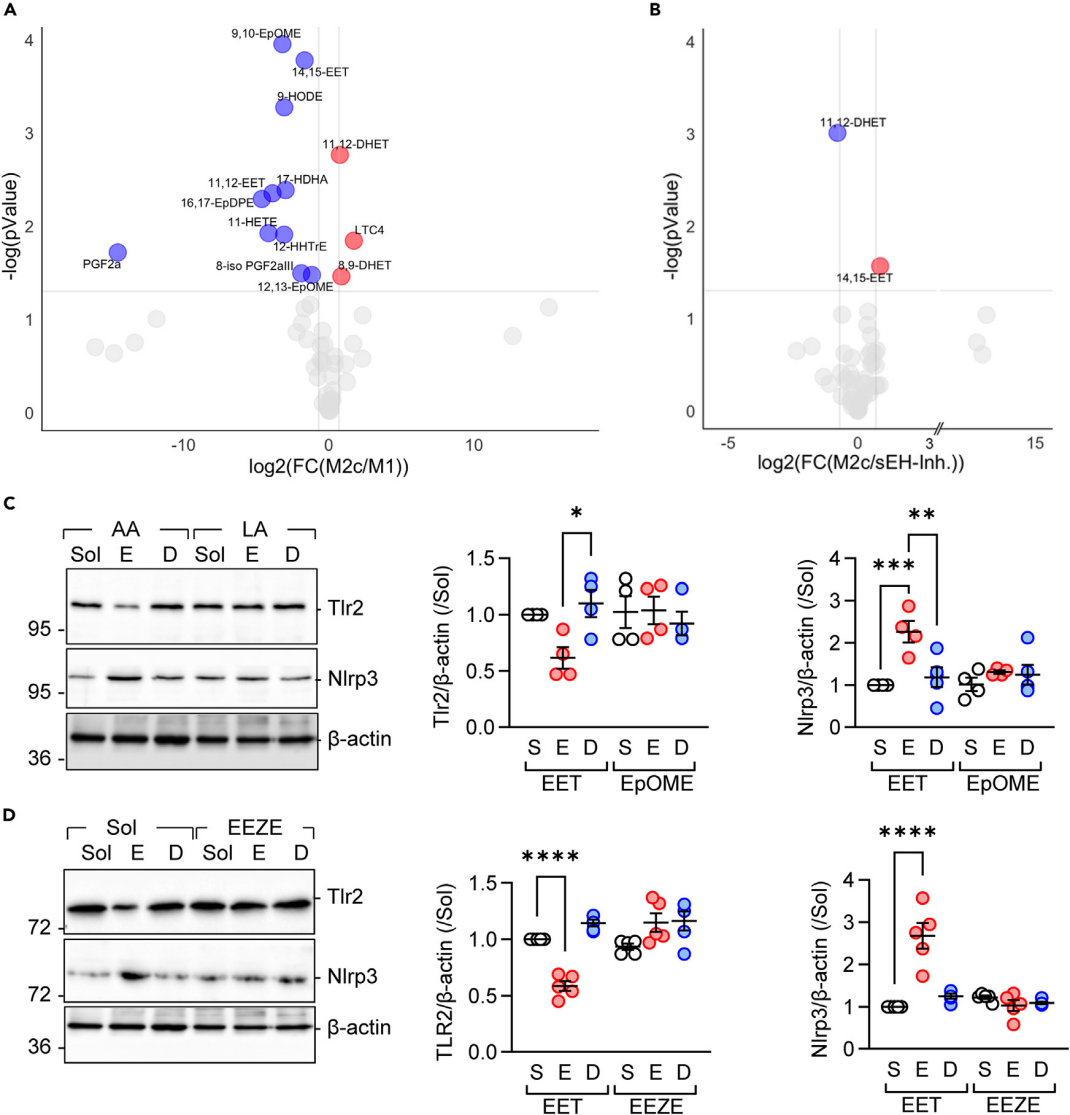
PUFA mediator profiling in human macrophages (A) PUFA mediator levels in M1- and M2c-polarized human-monocyte-derived macrophages; n = 6–7 donors per group. Red = significantly elevated in M2c, blue = significantly elevated in M1, gray = non-significant. (B) PUFA mediator levels in human macrophages polarized from M1 to M2c in the presence of solvent (Sol) or the sEH inhibitor (*t-*AUCB, 10 μmol/L); n = 6–7 donors per group. (C) Effect of the selected arachidonic acid (AA) and linoleic acid (LA) epoxide (E)-diol (D) pairs, i.e., 11,12-EET/DHET and 9,10-EpOME/DiHOME (all 1 μmol/L, 48 h) on Tlr2 and NLRP3 expression in M1-polarized human macrophages; *n* = 4 donors. (D) Impact of 11,12-EET (E) and 11,12-DHET (D; both 1 μmol/L, 48 h) on the expression of Tlr2 and Nlrp3. Experiments were performed in the absence and presence of the EET antagonist 14,15-epoxyeicosa-5(Z)-enoic acid (EEZE; 1 μmol/L); *n* = 5 donors. Error bars = SEM, (C and D) One-way ANOVA followed by Turkey’s multiple comparisons test. ∗*p* < 0.05, ∗∗*p* < 0.01, ∗∗∗*p* < 0.001, ∗∗∗∗*p* < 0.0001.

To determine which sEH substrate or product could contribute to the effects observed, epoxide and diol levels were assessed in M1- and M2c-polarized human macrophages. In M1 macrophages, levels of prostaglandin (PG) F2α and 8-iso-PGF2α were increased as expected, reflecting the induction of cyclooxygenase and oxidative stress (Figure 5A). There were also a number of changes in PUFA epoxides and diols, particularly the linoleic acid epoxides 9,10- and 12,13-epoxyoctadecaenoic acid (EpOME) and the arachidonic acid epoxides 11,12- and 14,15-EET. The latter effects were largely consistent with a previous report.^44^ M1 to M2c repolarization induced a pronounced increase in 8,9- and 12,13-dihydroxyeicosatrienoic acids (DHETs), which are generated from the corresponding EETs by the sEH. A comparison of M2c macrophages incubated without and with the sEH inhibitor *t*-AUCB revealed significant changes in only 11,12-DHET and 14,15-EET (Figure 5B). As 11,12-EET/DHET and 9,10-EpOME/DiHOME were the most consistently altered mediators, we determined whether they were able to reproduce the effects of sEH deletion on Tlr2 and Nlrp3 expression. Only 11,12-EET was able to decrease the expression of Tlr2 and increase the expression of Nlrp3, i.e., to reproduce the effects seen in sEH^−/−^ mice (Figure 5C). The actions of 11,12-EET were not observed in the presence of the previously characterized EET antagonist, 14,15-epoxyeicosa-5(*Z*)-enoic acid^45^ (Figure 5D).

### Crosstalk between sEH and TGF-β signaling

**Figure 6:**
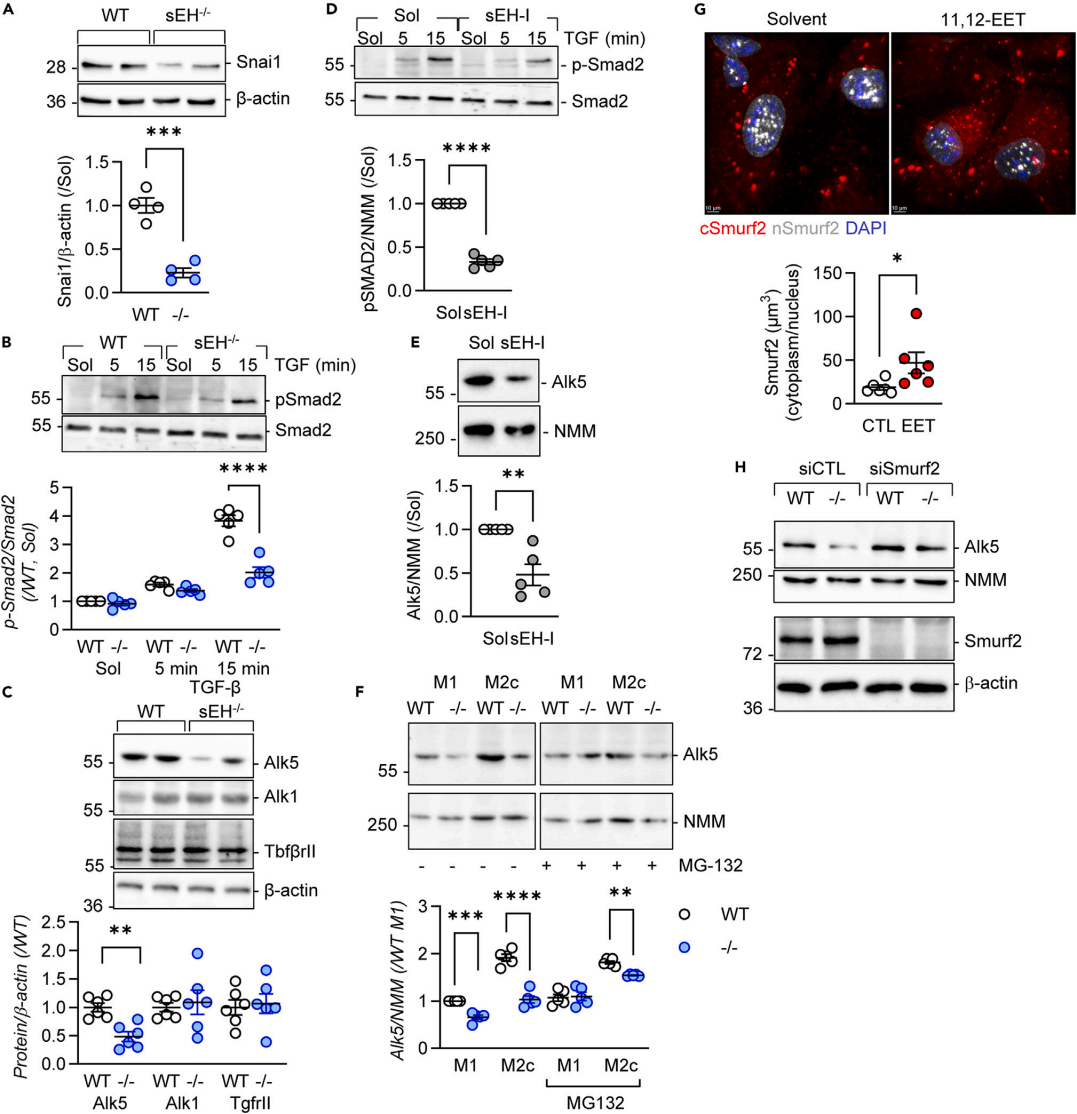
Effect of altered sEH expression on TGF-β signaling (A) Snai1 expression in M2c macrophages from wild-type (WT) and sEH^−/−^ (−/−) mice; *n* = 4 animals per group. (B) TGF-β-stimulated phosphorylation of Smad2 in M1-polarized macrophages; *n* = 5 animals per group. (C) Expression of Alk5, Alk1, and TgfβrII in M1-polarized macrophages; *n* = 6 animals per group. (D and E) Impact of sEH inhibition (sEH-I) on the TGF-β (15 min)-induced phosphorylation of SMAD2 phosphorylation in M1 macrophages from wild-type mice (D) and expression of Alk5 (e); *n* = 5 animals per group. (F) Alk5 expression in M1 and M2c macrophages in the absence and presence of MG132; *n* = 5 mice per group. The image shows non-adjacent lanes from the same blot with the excised section indicated by a break. (G) Nuclear (n) and cytoplasmic (c) levels of Smurf2 in cells treated with Solvent (0.1% DMSO) or 11,12-EET (3 mmol/L) scale bar = 10 μm; *n* = 6 animals per group. (H) Consequence of siRNA-mediated Smurf2 knockdown on Alk5 expression in M1 macrophages; comparable results were obtained using an additional three animals per group. Error bars = SEM, A, C, D, E, and G) Student’s t test; (B and F) two-way ANOVA followed by Sidak’s multiple comparisons test. ∗*p* < 0.05, ∗∗*p* < 0.01, ∗∗∗*p* < 0.001, ∗∗∗∗*p* < 0.0001.

Given that M2c polarization was induced by adding TGF-β to M1-polarized macrophages, potential links between sEH and TGF-β1 signaling were assessed. Interestingly, the expression of *Snai1* was consistently decreased in M2c macrophages from sEH^−/−^ mice (see Figure 3B) and was confirmed at the protein level (Figure 6A). Moving upstream in the signaling pathway, the TGF-β1-induced phosphorylation of Smad2 was found to be consistently lower in M1-polarized macrophages from sEH^−/−^ mice (Figure 6B). Next, we assessed the expression of TGF-β receptors, and although levels of TgfβrII and Alk1 were comparable in cells from both genotypes, the expression of Alk5 was significantly reduced in sEH-deficient macrophages (Figure 6C). Similar effects on the phosphorylation of Smad2 and the expression of Alk5 were observed after treating macrophages from wild-type mice with an sEH inhibitor (Figures 6D and 6E).

TGF-β can regulate the expression of its receptors,^46^ and although it increased Alk5 expression in M2c macrophages from wild-type mice, it was without effect in cells from sEH^−/−^ mice (Figure 6F). However, we detected no difference in Alk5 mRNA expression in our RNA-seq studies, implying that the effect may be regulated at the level of protein degradation. Indeed, inhibiting proteasomal degradation using MG132 normalized Alk5 levels so that they were comparable in sEH-expressing and -deficient cells. Little is known about the mechanism(s) regulating Alk5 expression but a role has been attributed to Smad ubiquitination regulatory factor 2 (Smurf2); an E3 ubiquitin ligase that has a major impact on TGF-β signaling dynamics.^47^ Because the activation of Smurf2 has been linked to its translocation out of the nucleus to form a complex with inhibitory Smads and ubiquitinate its targets (for review see^48^), we assessed the consequences of treating polarized macrophages with 11,12-EET. In untreated cells, Smurf2 was localized in the nucleus as well as in cytosolic aggregates, and treating cells with 11,12-EET promoted its nuclear to cytosolic translocation (Figure 6G). Consistent with its role in the degradation of Alk5, targeting Smurf2 using a siRNA approach slightly increased Alk5 levels in cells from wild-type mice and markedly increased its expression in sEH-deficient cells (Figure 6H).

## Discussion

The results of our study suggest that an intimate relationship exists between TGF-β and sEH. Indeed, the expression of sEH was increased by TGF-β and dependent on the activation of Alk5 and the binding of phosphorylated Smad2 to the sEH promoter. Moreover, the expression and activity of sEH had a clear impact on TGF-β signaling, as Alk5 expression was decreased in cells from sEH^−/−^ mice or from wild-type mice treated with an sEH inhibitor. The latter effect could be attributed to the accumulation of the sEH substrate 11,12-EET, as it elicited the proteolytic degradation of Alk5 by initiating the cytosolic translocation of the E3 ligase, Smurf2, which can ubiquitinate the receptor. These steps seem to make up an autocrine loop between 11,12-EET and TGF-β1 that determines macrophage function.

Several studies have investigated the anti-inflammatory effects of sEH inhibition or deletion, focusing mainly on neutrophil and macrophage recruitment.^5^^,^^20^^,^^21^ There have been hints that the enzyme also affects the resolution process. For example, the inhibition of CYP enzymes upstream of the sEH has been reported to reduce inflammatory resolution by altering Ly6c+ monocyte infiltration and thus reducing macrophage efferocytosis.^44^ We therefore decided to re-evaluate the role of the sEH in inflammation by monitoring the consequences of its deletion in a self-resolving model of peritonitis. As reported by others,^4^^,^^5^^,^^20^^,^^21^ we observed that the acute inflammatory response was attenuated in sEH^−/−^ mice, as significantly fewer neutrophils were recovered in the peritoneal lavage. The model we chose is normally self-resolving over 3–6 days but sEH^−/−^ mice showed incomplete/delayed resolution of inflammation *in vivo*. This was apparent as a failure to replenish the numbers of resident macrophages and dendritic cells and by incomplete differentiation of macrophages to a pro-resolving phenotype. However, since sEH inhibition and deletion attenuates both progenitor cell proliferation and myeloid cell recruitment from the bone marrow,^31^ human-blood-derived and murine bone-marrow-derived macrophages were used to assess the role of the sEH in regulating macrophage function in more detail.

Looking more closely at macrophage subtypes, particularly the well-characterized M1 and M2a phenotypes induced by pro- and anti-inflammatory stimuli,^49^ we found that the sEH was expressed but that its levels were comparable in both states. However, there was a pronounced increase in sEH expression when pro-resolving M2c macrophages were generated by re-polarizing classically activated (M1) macrophages with TGF-β1. This protocol was chosen because although the signals and events that determine the progression from the M1 to the M2c phenotype have not been fully elucidated, TGF-β has been ascribed a crucial role.^50^ The TGF-β signaling pathway is complex, with different signaling cascades activated by different combinations of receptors.^51^ We found TGF-β to have a profound impact on the expression of the sEH that was attributable to the activation of the TGF-β type I receptor Alk5, the phosphorylation of Smad2, and its binding to the sEH promoter. This was confirmed independently by assessing TGF-β-induced changes in sEH levels in mice lacking the Alk5 antagonist SMOC1. In SMOC1^+/−^ mice, the expression of the sEH was almost double than that seen in their wild-type littermates. Intriguingly, sEH levels increased to a greater extent in M2c macrophages from female mice and humans. Sex-specific differences in sEH expression have been reported previously, and the higher sEH levels in male mice made them more susceptible to the anti-hypertensive actions of sEH inhibition.^33^ It was later shown that the female-specific downregulation of sEH expression is driven by estrogen-dependent methylation of *Ephx2*, which significantly reduces the binding of Sp1, AP-1, and NF-κB to the promoter.^34^ This appears to be overcome by TGF-β/Smad2, as sEH levels were comparable in M2c-polarized macrophages from males and females.

M2c macrophages are crucial for the resolution of inflammation, as they possess the phagocytic/efferocytic activity required to remove cellular debris. Consistent with the delayed resolution phenotype observed *in vivo*, sEH-deficient macrophages were clearly less efficient at phagocytosing zymosan and ox-LDL than cells from wild-type littermates. RNA-sequencing was used to assess changes in gene expression in M2c macrophage that were dependent on sEH activity. In the absence of sEH, the expression of several scavenger receptors that play an important role in phagocytosis and efferocytosis were decreased. These changes fit well with the functional phenotype, and similar effects were seen in macrophages from wild-type mice treated with an sEH inhibitor. Furthermore, in M2c-polarized macrophages from sEH^−/−^ mice, overexpression of a wild-type, but not an enzymatically inactive, sEH mutant increased Tlr2 and Mrc1 levels. To determine whether the accumulation of an sEH substrate or the absence of a product could explain the consequences of sEH deletion on macrophage function, we determined epoxide/diol levels in M1- and M2c-polarized human macrophages. This revealed changes in 11,12-EET/DHET, 14,15-EET/DHET, and 9,10-EpOME/DiHOME. However, only 11,12-EET was able to decrease the expression of Tlr2 and increase the expression of Nlrp3 to reproduce the effects seen in M1-polarized macrophages from sEH^−/−^ mice.

Given that M2c polarization was induced by the addition of TGF-β to M1-polarized macrophages, potential links between sEH and TGF-β1 signaling were analyzed more closely. The fact that the expression of Snai1 was clearly and consistently downregulated in M2c macrophages from sEH^−/−^ mice at both RNA and protein levels hinted that in addition to TGF-β1 regulating sEH expression, the activity of the sEH has a marked impact on TGF-β signaling. Circumstantial evidence for a close link between the CYP-sEH and TGF-β pathways already exists, as EETs have been reported to inhibit the activation of murine fibroblasts by blocking Smad2/3 signaling.^52^ It has also been reported that sEH inhibitors attenuate TGF-β signaling in a model of pulmonary fibrosis,^53^ and dual inhibition of cyclooxygenase and sEH protects against liver fibrosis^54^ and the TGF-β-driven development of epithelial to mesenchymal transformation in the lung.^55^ Indeed, moving up the signaling cascade, we found that the acute TGF-β1-induced phosphorylation of Smad2 in M1-polarized macrophages was also attenuated in sEH-deficient M2c macrophages. This hinted that metabolites of the CYP-sEH pathway could affect either the expression of TGF receptors or their activity and led to the identification of Alk5 as a TGF-β receptor whose expression was regulated in an sEH-dependent manner.

Not a lot is known about the mechanisms that regulate Alk5 expression in macrophages. However, its expression is increased by stimuli such as LPS, IFN-γ,^56^ ox-LDL,^57^ and TGF-β.^46^ Our experiments also demonstrated that adding TGF-β1 to macrophages increased Alk5 expression but only in cells expressing sEH. Alk5 levels can be altered at the level of gene expression as well as by protein degradation.^58^ As we did not observe consistent changes in Alk5 at the RNA level, we investigated the effect of preventing its proteosomal degradation and successfully rescued Alk5 levels. This led us to focus on the E3 ligase, Smurf2, which ubiquitinates TGF-β receptors, thus promoting their proteasomal degradation.^59^ TGF-β stimulation has been shown to cause Smurf2 to translocate from the nucleus to the cytosol where it forms a complex with inhibitory Smads and ubiquitinates type I TGF-β receptors.^47^ The subsequent proteasomal degradation of these proteins thus attenuates TGF-β signaling (for review see^48^). Interestingly, we found that the sEH substrate, 11,12-EET, effectively induced the cytoplasmic translocation of Smurf2 and that downregulating Smurf2 increased Alk5 expression in sEH^−/−^ to levels observed in wild-type cells. Our findings go a long way toward explaining a previously suggested link between Smurf2 and the CYP-sEH axis, based on the interaction of CYP2J3 with flavin-containing monooxygenase 2 in cardiac fibroblasts that was proposed to determine the intracellular localization of Smurf2.^60^ Although the ability of PUFA mediators to initiate the ubiquitination and degradation of proteins has not been studied in detail, this may represent a broader mechanism of action for these lipid mediators. For instance, CYP2J2 was reported to promote the ubiquitination of NLRX1,^61^ and we previously observed that 11,12-EET induced the activation of PPAR-γ prior to inducing its proteasomal degradation.^62^ Whether the latter effects involve Smurf2 or an alternative E3 ligase remains to be determined. Another open question is how 11,12-EET was able to initiate its effect. There are numerous lines of evidence hinting that a membrane receptor, most probably a G-protein-coupled receptor, exists for 11,12-EET, but a specific structure has not yet been definitively identified.^63^ In this study, the finding that an EET analogue that antagonizes the effects of EETs in several systems^64^ was able to prevent the changes in Tlr2 and Nlrp3 expression suggests that the effects of 11,12-EET may be mediated through activation of an EET receptor.

Taking all of our data together, we conclude that not only is the expression of sEH in pro-resolving macrophages controlled by TGF-β, but that the activity of the enzyme determines TGF-β signaling and macrophage repolarization to a pro-resolving phenotype. At the molecular level, the regulation of Alk5 levels by 11,12-EET can account for the effects observed *in vitro* and *in vivo*. This unexpectedly intimate relationship seems to be an integral part of an autocrine loop between 11,12-EET and TGF-β1 that determines cell function. Such a mechanism may have wide-reaching consequences and contribute to the consequences of manipulating sEH expression on processes such as angiogenesis, atherosclerosis, and organ fibrosis.^1^

### Limitations of the study

Despite the data showing a clear link between a PUFA epoxide and TGF-β signaling, there are limitations in the current study that warrant consideration. Firstly, the mice studied carried a constitutive global deletion in the Ephx2 gene, rather than an inducible cell-specific knockout, which may be associated with compensatory changes over time. Because of this, key experiments were repeated with and without an sEH inhibitor. Secondly, the exact mechanism of action linking 11,12-EET with Smurf2 was not identified. There are data linking the actions of the epoxide with a membrane receptor but this has yet to be identified.

## Resource availability

### Lead contact

Further information and requests for resources should be directed to and will be fulfilled by the lead contact, Dr. Ingrid Fleming (Fleming@em.uni-frankfurt.de).

### Materials availability

This study did not generate new unique reagents.

### Data and code availability

• Data: single-cell RNA-seq data have been deposited at GEO and are publicly available as of the date of publication. Accession numbers are listed in the key resources table. Original western blot images have been deposited at Mendeley and are publicly available as of the date of publication. The DOI is listed in the key resources table. Microscopy data reported in this paper will be shared by the lead contact upon request.
• Code: all original code has been deposited at Zenodo and is publicly available as of the date of publication. DOIs are listed in the key resources table.
• Additional information: any additional information required to reanalyze the data reported in this paper is available from the lead contact upon request.

## Acknowledgments

The authors are indebted to Orwa Barakat and Oliver Haun for expert technical assistance. This work was supported by the 10.13039/501100001659Deutsche Forschungsgemeinschaft (GRK 2336 TP5–Project ID 321115009; SFB1039/3 B6–Project ID 204083920, and the 10.13039/501100021703Cardio-Pulmonary Institute, EXC 2026, Project ID: 390649896). X.L. was supported by a grant from the China Scholarship Council and F.D.L. by a stipend from the 10.13039/501100020027Dr. Rolf M. Schwiete Stiftung (Project ID: 2021-025).

## Author contributions

X.L., designed the research and acquired and analyzed data; S.K., designed the research and acquired and analyzed data; F.D.L., acquired and analyzed data; U.U., acquired and analyzed data; R.P., acquired and analyzed data; J.H., provided feedback, edited the text; T.F., acquired and analyzed data; S.G., acquired and analyzed data; A.W., designed and interpreted the FACS data; I.F., designed the research, analyzed the data, acquired funding, and wrote the manuscript.

## Declaration of interests

The authors declare no competing financial interests.

## STAR★Methods

### Key resources table

**Table:** 

REAGENT or RESOURCE	SOURCE	IDENTIFIER
**Antibodies**		

CD45-VioBlue	Milteny, Bergisch Gladbach, Germany	#130-102-430; RRID: AB_2659925
CD31-PE-Cy7	eBioscience, Frankfurt, Germany	#25-0311-82; RRID: AB_2716949
MHC class II-APC-eFluor-780	ThermoScientific, Darmstadt, Germany	#47-5321-81; RRID: AB_1548783
CD19-APC-Fire750	Biolegend, Koblenz, Germany	#115558; RRID: AB_2572120
γδ TCR-APC	Biolegend, Koblenz, Germany	#118116; RRID: AB_1731813
CD8-BV650	Biolegend, Koblenz, Germany	#100742; RRID: AB_2563056
GITR-FITC	Biolegend, Koblenz, Germany	#126308; RRID: AB_1089125
F4/80-PE-Cy7	Biolegend, Koblenz, Germany	#123114; RRID: AB_893478
Ly6G-APC-Cy7	Biolegend, Koblenz, Germany	#127624; RRID: AB_10640819
Ly6C-PerCP/Cyanine5.5	Biolegend, Koblenz, Germany	#128012; RRID: AB_1659241
CD11b-BV605	Biolegend, Koblenz, Germany	#101237 RRID: AB_11126744
CD3-PECF594	BD Bioscience, Heidelberg, Germany	#562286; RRID: AB_11153307
CD4-BV711	BD Bioscience, Heidelberg, Germany	#563050; RRID: AB_2737973
NK1.1-BV510	BD Bioscience, Heidelberg, Germany	#563096; RRID: AB_2738002
p-SMAD2 (Ser465/467)	Cell Signaling Technology Europe, Leiden, The Netherlands	# 3108; RRID: AB_10117720
SMAD2	Cell Signaling Technology Europe, Leiden, The Netherlands	# 5339; RRID: AB_10858226
p-SMAD1/5	Cell Signaling Technology Europe, Leiden, The Netherlands	# 9516; RRID: AB_10121682
SMAD1	Cell Signaling Technology Europe, Leiden, The Netherlands	# 6944; RRID: AB_10860070
ALK5	abcam, Cambridge, UK	# ab31013; RRID: AB_778352
TGFRII	abcam, Cambridge, UK	ab186838; RRID: AB_2728775
TLR2	abcam, Cambridge, UK	# ab16894; RRID: AB_443530
CD206	NOVUS Biologicals, Wiesbaden Nordenstadt, Germany	# NBP1-90020; RRID: AB_11036359
NLRP3	NOVUS Biologicals, Wiesbaden Nordenstadt, Germany	# NBP2-12446; RRID: AB_2750946
ALK1	OriGene, Rockville, USA	# AP01172-PU-N; RRID: AB_1609796
β-actin	Linaris, Eching, Germany	# MAK6019
sEH	ProteinTech, Martinsried, Germany	Custom made
goat anti-rabbit	Merck, Darmstadt, Germany	# 401393; RRID: AB_437797
goat anti-mouse	Merck, Darmstadt, Germany	# 401253; RRID: AB_437779
goat anti-rat	Merck, Darmstadt, Germany	#401411; RRID: AB_212007
rabbit anti-goat	Merck, Darmstadt, Germany	# 401515; RRID: AB_437816
F4/80	eBioscience, CA, USA	#14-4801-85; RRID: AB_467559
Smurf2	Thermo Fisher, CA, USA	MA5-45004; RRID: AB_2931458
Alexa Fluor-546	Thermo Fisher Scientific, Dreieich, Germany	# A10040; RRID: AB_2534016
Alexa Fluor-488	Thermo Fisher Scientific, Dreieich, Germany	# A32790; RRID: AB_2762833
Alexa Fluor-633	Thermo Fisher Scientific, Dreieich, Germany	# A-21070; RRID: AB_2535731

**Chemicals, peptides, and recombinant proteins**		

RPMI 1640 Medium	Gibco, Invitrogen, Darmstadt, Germany	# 2242222
Minimum Essential Medium	Gibco, Invitrogen, Darmstadt, Germany	# 2209288
MEM non-essential amino acids solution	Gibco, Invitrogen, Darmstadt, Germany	# 11140050
MEM Vitamin Solution	Gibco, Invitrogen, Darmstadt, Germany	# 11120052
Fetal bovine serum	Gibco, Invitrogen, Darmstadt, Germany	#10270-106
14,15-epoxyeicosa-5(Z)-enoic acid	Cayman Chemical, Tallinn, Estonia	#10004946
(±)11,(12)-epoxy-5Z,8Z,14Z-eicosatrienoic acid	Cayman Chemical, Tallinn, Estonia	# 50511
(±)11,12-dihydroxy-5Z,8Z,14Z-eicosatrienoic acid	Cayman Chemical, Tallinn, Estonia	#51511
(±)9,10-epoxy-12Z-octadecenoic acid	Cayman Chemical, Tallinn, Estonia	#52400
(±)9,10-dihydroxy-12Z-octadecenoic acid	Cayman Chemical, Tallinn, Estonia	#53400
Macrophage colony-stimulating factor	peprotech, Hamburg, Germany	#315-02
Granulocyte macrophage colony-stimulating factor	peprotech, Hamburg, Germany	# 315-03)
human interferon γ	peprotech, Hamburg, Germany	# 300-02
murine IFN-γ	peprotech, Hamburg, Germany	# 315-05
murine interleukin 4	peprotech, Hamburg, Germany	# 214-14
human IL-4	peprotech, Hamburg, Germany	# 200-04
human transforming growth factor-β1	peprotech, Hamburg, Germany	# 100-21
pHrodo Red Zymosan Bioparticles	Invitrogen, Darmstadt, Germany	# P35364
DiI-oxLDL	Invitrogen, Darmstadt, Germany	# L34358

**Critical Commercial**		

Interleukin 1β ELISA	Bio-Techne GmbH, Wiesbaden-Nordenstadt, Germany	

**Experimental models: Organisms/strains**		

Mouse: C57BL/6N mice	Charles River, Sulzfeld, Germany	
Mouse: B6D2-Smoc1/JtakRbrc	RIKEN BioResource Center, Tsukuba, Japan	
Mouse: Ephx2^tm1.1Arte^ x Gt(ROSA)26Sortm16(Cre)Arte	TaconicArtemis GmbH Cologne, Germany	

**Oligonucleotides**		

	See Table S1 for PCR primers	

**Software and algorithms**		

Prism	GraphPad Software Inc., Boston, USA	9.0.1; RRID: SCR_002798
Imaris	Bitplane AG, Zürich, Switzerland	10.0; RRID: SCR_007370
Biorender	Used for preparation of the graphical abstract	Licence # OO272BWW7T & SU272BWWB8; RRID: SCR_018361

**Deposited data**		

RNA sequencing data	Gene Expression Omnibus (GEO; www.ncbi.nlm.nih.gov/geo) repository	GSE273900
Uncropped Western blots	https://data.mendeley.com/datasets/nz789jbbxc/1	https://doi.org/10.17632/nz789jbbxc.1
Code for analysis of RNA-seq	https://zenodo.org/records/13341724	https://doi.org/10.5281/zenodo.13341724

### Experimental model and study participant details

#### Animals

C57BL/6N mice (6–8 weeks old) were from Charles River (Sulzfeld, Germany), SMOC1^+/−^ (B6D2-Smoc1<Tn(sb-lacZ,GFP)PV384Jtak>/JtakRbrc) mice were from the RIKEN BioResource Center (Tsukuba, Japan) and sEH^−/−^ mice were generated as described.^65^ Age- and strain-matched mice (littermates) were used throughout, with most experiments being performed on cells from females. For the isolation of bone marrow, mice were sacrificed using 4% isoflurane in air and subsequent exsanguination.

#### Zymosan-induced peritonitis

To induce a self-resolving inflammation, zymosan-A (10 mg/kg in 200 μL PBS, i.p.) was injected female wild-type and sEH^−/−^ mice (8–12 weeks old). One, 3, and 6 days after zymosan injection, chemokines and cells in the peritoneum were isolated via lavage using 3 mL ice-cold PBS, as described.^66^ For determination of the composition of immune cells in the lavage, cells were stained with different antibodies and analyzed using flow cytometry (BD, LSRFortessa). Data were analyzed using FlowJo software (version 10.6.2). In some experiments the expression of inflammatory markers was determined by RT-qPCR.

#### Ethics statement

All animals were housed in conditions that conform to the Guide for the Care and Use of Laboratory Animals published by the U.S. National Institutes of Health (NIH publication no. 85-23). Both the University Animal Care Committee and the Federal Authority for Animal Research at the Regierungspräsidium Darmstadt (Hessen, Germany) approved the study protocol (FU2009, 2021).

### Method details

#### Monocyte isolation and culture

##### Human monocytes

Human monocytes were isolated from peripheral blood from healthy donors (DRK-Blutspendedienst Baden-Württemberg-Hessen, Frankfurt) as described,^67^ and further differentiated to naive (M0) macrophages in RPMI supplement with 3% human plasma for 7 days. Macrophages were polarized to M1 macrophages (100 ng/mL LPS and 20 ng/mL hIFNγ,12 h), M2a macrophages (20 ng/mL hIL-4, 24 h) and M2c macrophages from M1 macrophages by TGF-β1 (10 ng/mL) for 48 h.

##### Murine monocytes

Murine monocytes were isolated from bone marrow of 8–10 week old mice. Monocytes were differentiated to naive (M0) macrophages in RPMI 1640 medium containing 8% heat inactivated FCS supplemented with M-CSF (15 ng/mL) and GM-CSF (15 ng/mL) for 7 days. Thereafter M0 macrophages were polarized to M1 macrophages (LPS, 10 ng/mL and murine IFN-γ, 1 ng/mL, and 12 h) or M2a macrophages (murine IL-4, 20 ng/mL, 24 h). M2c macrophages were generated by treating M1 macrophages with TGF-β1 (10 ng/mL) for 24 or 48 h.

#### Fluorescence-activated cell sorting (FACS) analyses

Single cell suspensions were stained with florescent antibodies and analyzed by flow cytometry using an LSRII Fortessa cell analyzer (BD Biosciences, Heidelberg, Germany). Antibodies were titrated to determine optimal concentrations. For single-color compensation CompBeads (BD Bioscience, Heidelberg, Germany) were used to create multicolor compensation matrices. Cells were blocked with 2% FcR Binding Inhibitor (Milteny, Bergisch Gladbach, Germany) in PBS for 10 min on ice for 10 min. Afterward, the cells were stained with different antibodies for 10 min. For gating, fluorescence minus one controls (FMO; containing all antibodies, minus one of them, for gating cells that express the omitted antigen) were used. Instrument calibration was controlled daily using Cytometer Setup and Tracking Beads (BD Bioscience, Heidelberg, Germany). Gating is outlined in Figure S6. Leukocyte categories were defined as follows: neutrophils (CD11b+ Ly6G+), monocytes (CD11b+ F4/80low Ly6G- Ly6C+/−), macrophages (CD11b+ F4/80+ Ly6G-), NK Cells (NK1.1+ CD3ε-), NK T cells (NK1.1+ CD3ε+), dendritic cells (CD11c+ CD11b+ Ly6C- F4/80-), T cells (CD11b-, CD19^−^, CD3ε+).

#### Interleukin 1β ELISA

The levels of interleukin 1β were determined in cell supernatant by ELISA (Bio-Techne GmbH, Wiesbaden-Nordenstadt, Germany) according to the manufacturer’s protocol.

#### Immunoblotting

Cells were lysed in RIPA lysis buffer (50 mmol/L Tris/HCL pH 7.5, 150 mmol/L NaCl, 10 mmol/L NaPPi, 20 mmol/L NaF, 1% sodium deoxycholate, 1% Triton X-100 and 0.1% SDS) enriched with protease and phosphatase inhibitors. Detergent soluble proteins were recovered by centrifugation, separated by SDS-PAGE and subjected to Western blotting as described.^68^ After blocking in Tris-buffered saline containing 0.3% Tween 20 and 3% BSA, membranes were exposed to primary antibodies (overnight 4°C) and horseradish peroxidase-conjugated secondary antibodies (2 h, room temperature). Protein bands were visualized using Lumi-Light plus Western blotting substrate (Roche, Mannheim, Germany) and captured by an image acquisition system (Fusion FX7; Vilber-Lourmat, Torcy, France).

#### RNA isolation and quantitative real-time PCR (RT-qPCR)

Total RNA was extracted from cells and tissues using QIAgen RNeasy kits (Invitrogen, Carlsbad, CA, USA) according to the manufacturer’s protocol and RNA (1 μg) was used for reverse transcription (RTase SuperScript IV, Invitrogen). Primers (BioSpring GmbH, Germany) were designed using an online tool, primer3 (https://primer3.ut.ee), and the cDNA was amplified using SensiFAST SYBR No-ROX kit (GENTAUR GmbH, Aachen, Germany) and a real-time thermal cycler (BMS, Queensland, Australia). All RNAs were normalized to 18S rRNA. Quantitative PCR was performed using SYBR green master mix (Biozym, Hessisch Oldendorf, Germany) and appropriate primers (Table S1) in a MIC-RUN quantitative PCR system (Bio Molecular Systems, Upper Coomera, Australia). The relative RNA amounts were calculated using the 2^−ΔΔCT^ method with 18S RNA as a reference.

##### CHIP-qPCR

Crosslinking and isolation of nuclei was performed with the truCHIP chromatin shearing kit (Covaris, USA) according to the manufacturer’s protocol. Thereafter, chromatin immunoprecipitation was performed as described.^69^ Thereafter, the binding of SMAD2 to the sEH promoter was monitored via qPCR using the primers listed in Table S1.

#### RNA sequencing

Total RNA was isolated from macrophages by using RNeasy Micro kit (Qiagen, Hilden, Germany) based on manufacturer’s instructions and as described.^62^ Thereafter, RNA concentrations were determined (NanoDrop ND-1000, Thermo Fisher Scientific, Dreieich, Germany; λ 260 nm) and RNA (1 μg) was used as input for SMARTer Stranded Total RNA Sample Prep Kit - HI Mammalian (Takara Bio, Kyoto, Japan). Trimmomatic version 0.39 was employed to trim reads after a quality drop below a mean of Q20 in a window of 20 nucleotides and keeping only filtered reads longer than 15 nucleotides.^70^ Reads were aligned versus Ensembl mouse genome version mm10 (Ensembl release 101) with STAR 2.7.10a.^71^ Aligned reads were filtered to remove: duplicates with Picard 2.25.5 (Picard: A set of tools (in Java) for working with next generation sequencing data in the BAM format), multi-mapping, ribosomal, or mitochondrial reads. Gene counts were established with featureCounts 2.0.2 by aggregating reads overlapping exons on the correct strand excluding those overlapping multiple genes.^72^ The raw count matrix was normalized with DESeq2 version 1.44.0.^73^ Contrasts were created with DESeq2 based on the raw count matrix. Genes were classified as significantly differentially expressed at average count >5, multiple testing adjusted *p*-value <0.05, and −0.585 < log2FC > 0.585. The Ensemble annotation was enriched with UniProt data.^74^ The principal component analysis, volcano plots and pathway enrichment analysis were generated using R with the following packages: pheatmap version 1.0.12 and ggplot2 version 3.5.0.^75^ The code is available upon request. RNA-seq data have been deposited with the Gene Expression Omnibus (GEO; www.ncbi.nlm.nih.gov/geo) repository and are publicly available as of the date of publication. Accession numbers are listed in the key resources table.

#### Phagocytosis assays

Murine macrophages were incubated with either pHrod Red Zymosan Bioparticles (10 μg/mL, Invitrogen) or dil-oxo-LDL (2.5 μg/mL; Thermofisher Scientific, Darmstadt, Germany) in RPMI medium supplemented with 0.1% BSA and phagocytosis was monitored over 24 h using an automated live cell imaging system (IncuCyte; Sartorius, Göttingen Germany).

#### HEK cell culture

HEK-293 cells were obtained from the American Type Culture Collection (LGC Standards, Wesel, Germany) and cultured in MEM containing 8% heat inactivated FCS, gentamycin (25 μg/mL), sodium pyruvate (1 mmol/L) and non-essential amino acids (Thermo Fisher Scientific, Dreieich, Germany). Cells were maintained in a humidified incubator at 37°C containing 5% CO_2_.

##### sEH promoter activity

The luciferase activity assay was performed as described previously.^35^ Briefly, HEK cells were seeded into a 24-well plates (Falcon) at a density of 5x10^4^ cells/well. After 24 h, cells were then transfected with 200 ng of plasmid carrying the human sEH promoter (−374/+28, −974/+28, −1474/+28, −2974/+28, −4174/+28, −4774/+28, −5974/+28 bp) using Lipofectamine 2000 according to the manufacturers protocol. For each transfection, 4 ng of a control plasmid (pRL-CMV expressing renilla luciferase) was used as an internal control. After 24 h, the transfected cells treated with either solvent or TGF-β1 (10 ng/mL) for another 24 h. Cells were then harvested and lysed with 100 μL lysis buffer. Luciferase activity was measured using Dual-Glo Luciferase Reporter assay (Promega) and determined with PerkinElmer 2104 EnVision Multilabel Plate Readers (Waltham, MA, USA). Firefly luciferase activity was normalized against renilla luciferase activity.

#### sEH mutants

Adenoviral mediated expression of the wild-type sEH or a catalytically inactive sEH mutant (Tyr383 and Tyr466 were mutated to phenylalanine) was achieved as described.^76^ After overnight transduction cells were washed with medium and treated as described in the results section.

#### sEH activity assay

##### PHOME assay

Soluble epoxide hydrolase activity was determined using cytosolic cell lysates as reported.^77^ Briefly, reaction was performed with 5 μg protein at 37°C for 10 min in 100 μL of potassium phosphate buffer (100 mmol/L, pH 7.2) containing 0.1% BSA and fresh 0.25 mmol/L phenylmethylsulfonylfluoride, were added in a 96 well plate and then mixed with 50 μL of 40 μmol/L (3-phenyloxiranyl)acetic acid cyano(6-methoxynaphthalen-2-yl) methyl ester (PHOME) at 37°C. Fluorescence was recorded (excitation λ330 nm, emission λ465 nm) over 1 h using a plate reader (EnVision, PerkinElmer, Waltham, MA, USA).

*LC-MS/MS*: Murine macrophages were lysed in Triton lysis buffer (50 mmol/L Tris/HCl pH7.5, 150 mmol/L NaCl, 10 mmol/L NaPPi, 20 mmol/L NaF, 1%Triton X-100) and sEH activity determined as described,^76^ using a Sciex API4000 mass spectrometer operating in multiple reaction monitoring mode.

#### PUFA metabolite profiling

Samples were prepares, extracted and analyzed as reported previously.^78^ All samples and dilutions of the standards were spiked with internal deuterated standards: 8,9-DHET-d11, 11,12-DHET-d11, 14,15-DHET-d11, 9,10-DiHOME-d4, 12,13-DiHOME-d4, 5,6-EET-d11, 8,9-EET-d8, 11,12-EET-d8, 14,15-EET-d8, 9.10-EpOME-d4, 12,13-EpOME-d4, 5S-HETE-d8, 12S-HETE-d8, 15S-HETE-d8, 20-HETE-d6, 9S-HODE-d4, 13S-HODE-d4. Concentrations were determined by reference to the standards. Analyst 1.6.2 and MultiQuant 3.0 (both Sciex, Darmstadt, Germany), were used for data acquisition and analysis, respectively.

#### Immunohistochemistry

Cells cultivated on Ibidi chambers were fixed with 4% paraformaldehyde (PFA) for 15 min at room temperature. Thereafter, samples were washed three times in phosphate buffered solution (PBS) (10 min each). Cells were blocked and permeabilized with 0.3% Triton X-100 in PBS containing donkey serum (5%, for 1 h). Afterward cells were incubated overnight with primary antibodies (4°C). Thereafter, primary antibody samples were removed and samples were incubated with respective Alexa Fluor-conjugated secondary antibodies for 60 min at room temperature. Cells were then washed three times in PBS (10 min each) and corresponding cell nuclei were counterstained with DAPI (10 ng/mL: Thermo Fisher, CA, USA). In experiments in which the nuclear to cytosolic shuttling of Smurf2 was assessed, confocal z-stacks were analyzed with Imaris (Version 10.0, Bitplane AG, Zürich, Switzerland).In detail, after the individual fluorescent channels were processed with a Gaussian filter, both the volume of the nucleus and the Smurf signals were rendered. The quotient of the volume of the Smurf signal located either outside or inside the nucleus was calculated. The rendered volume of the nucleus was used as a mask to split the fluorescence channel of the Smurf signal into a nuclear or cytosolic part.

### Quantification and statistical analysis

The data are presented as mean ± SEM. Statistical analysis was performed using Prism 9.0.2 (GraphPad Software Inc., Boston, USA). Data were analyzed using Student’s t-test, one-way ANOVA followed by Tukey’s multiple comparisons test, or two-way ANOVA with Sidak’s multiple comparisons test, where appropriate. A P-value of less than 0.05 was considered statistically significant.
