# Supplementary material for: A regulatory loop involving the cytochrome P450-soluble epoxide hydrolase axis and TGF-β signaling
Source: iScience. 2024 Sep 16;27(10):110938. doi: 10.1016/j.isci.2024.110938 (PMC11466655; doi:10.1016/j.isci.2024.110938)
Supplement: Document S1. Figures S1–S6 and Table S1 [file mmc1.pdf]

iScience, Volume *27*

Supplemental information

A regulatory loop involving the cytochrome P450-soluble epoxide hydrolase axis and TGF-β signaling

Xiaoming Li, Sebastian Kempf, Fredy Delgado Lagos, Ürün Ukan, Rüdiger Popp, Jiong Hu, Timo Frömel, Stefan Günther, Andreas Weigert, and Ingrid Fleming

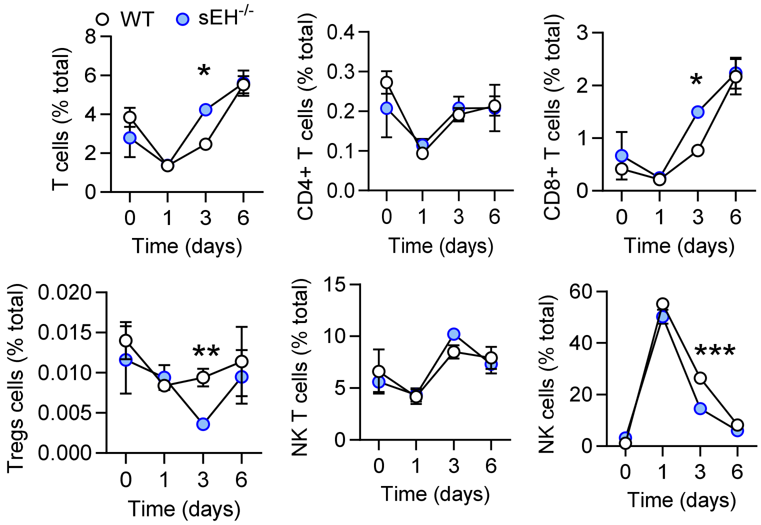

Supplementary Fig. 1. **Consequences of sEH deletion on the resolution of inflammation**. Related to Figure 1. Intraperitoneal cell populations isolated from wild-type (WT) and sEH$^{-/-}$ (-/-) mice up to 6 days after single intraperitoneal injection of zymosan. Shown are total T cells, CD4+ and CD8+ T cells, regulatory T cells (Tregs), natural killer T cells (NKT) and natural killer (NK) cells. n=5-6 animals per group. Two way ANOVA and Sidak's multiple comparisons test. *P<0.05, **P<0.01, ***P<0.001.

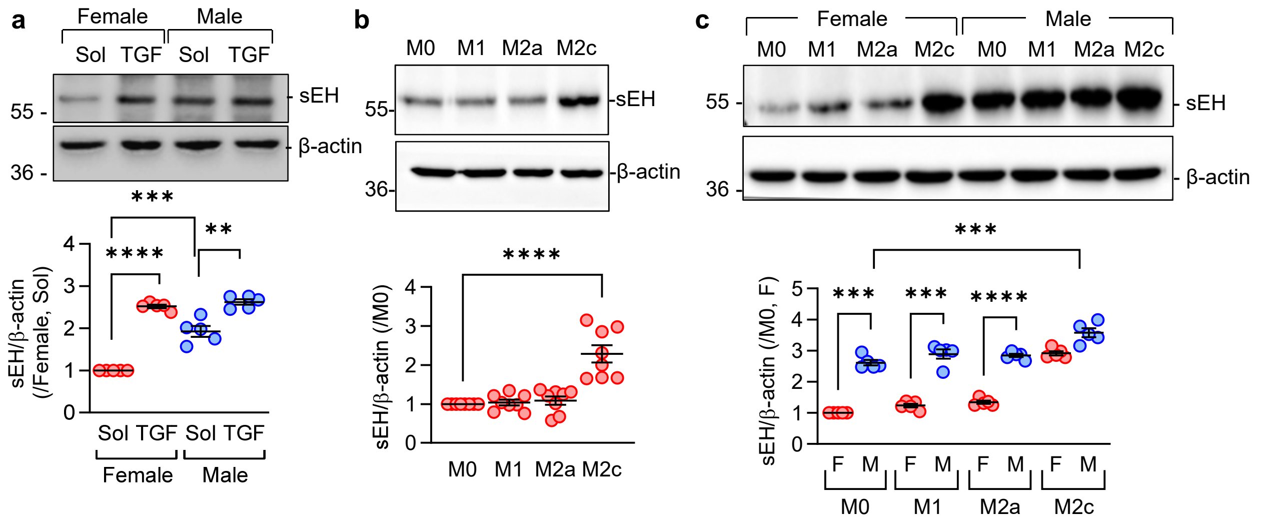

Supplementary Fig. 2. **sEH expression in polarized macrophages from female and male mice and humans**. Related to Figure 2. (**a**) Effect of solvent (Sol) and TGF-β1 (10 ng/mL, 48 hours) on sEH expression in M1 polarized macrophages from female and male mice; n=5 mice per group. (**b**) sEH expression in M0 (Sol), M1 (LPS /IFNγ, 12 hours), M2a (IL-4, 24 hours) and M2c (LPS/IFNγ and TGFβ1, 48 hours) polarized human macrophages from female donors; n=8 donors per group. (**c**) Expression of sEH in polarized human macrophages from male and female donors; n=5 subjects per group. a&c: two way ANOVA and Sidak's multiple comparisons test, b: one way ANOVA and Tukey's multiple comparisons test. **P<0.01, ***P<0.001, ****P<0.0001.

-6910-CATTTAGGAAAAAG**AGAC**TGGCCAAGATCACCAACAGGGTGATTGTAGATAAGAAGAGGAAGAGGTCCAAGGACCAACTTTTTATTTAGAGATT
AGGACAATGATGAGCCTGCAAAGGAAATCAGGCAGGAGCAGCCTGGGGGAGAGGATGAAAGCCAAGCGAGAAAGGATCTCAGAAGCCAGGA
AAGATGGCCTGCTAAGGAAGAGAGAGGAGCAGCTGGGCCAAAGGCTGCTGATAGGTCAGGTGCCACGAGGACTTCCATTGACCTTTGGATTT
AGCCATGTAGAGGTTTTTTTGGGATGTCGGCAAGATGACTTTCAGCAAGGTATAA**AGAC**AAAAGCATGATTTCAATGAGTTCAGGGGAGAATG
GGCCAAGAAGGCTTAGTGATAAAAAGTAGGAACAATTCTCTCAC**AGAC**TTTCTCTTTAAAAGAGAGAAATACGATGGTAACTGGAGGGAAATGT
GGGCTAAATATGTCATTTTTGTTGGTATGAGAATGAGATGATCCCATAGAGGAGTTAACCAACAATGTGGGAGATGTGGCATTGTTCTCTTGCC
TACAGGCGCACCGAATCTAATGCCTCAGTAGAAAGTTGGCCTCAGCTAAGAATGGTGGGGCCTGGACATCGTGGCAGCAGAGAATCTGCTTA
GGTGCAGGTAGGGGGGTTGACAGGGGGCTGGGATCCAGTGGAAGTTTTCTGTTTGTCCCAGTTTTCTCAAAGAAATAGGAAGAAAAGGTATT
GGCTGAGAGTAAGGATAGGGGAGAGAGTATTGGAGATGCAAGCAGAGAAGGGAGCTTACATA**GTCT**TCTAGGAGATTAGGAGAGAAAACGGA
TTAGAGAAGAACAGTAGAATTAGTGG**GTCT**TCAGGGACAATTCAGGAGCTGTGATGAGGACCAAGAAGGACAGCTGCCTGTCCTTGCCCAGC
AGCACAGGCAAGGAGGGGAGAGAACACTGAGCATTTGCAA**AGAC**TGCAGTGGGGGCCAGGCGTGCGGTGGGGGCCAGGCGTGCGGTGGG
GGCAGGCATGGTGGTTCACACCTGTCATCCCAACACTTTAGGAAGCCGAGGCAGGAGCTTGAAGCCAGGAGTTTG**AGAC**CAGCCTGAGCAA
CACAGCA**AGAC**CCCATCTCTACAAAAAATTTAAAAATTAGCCAAGCACAATGGCATATGCTTGTA**GTCT**GAGCTATTCAGGAGGCTAAGGTGGG
AGGATCGCTTAAGTTCAGGAGTTCGAGGTTACAGTGATGTAGGATTTTTCTTCTCAATCACTTTGCAAGCTGAGGACTTCTGGCCAGCAACACC
CCACCCAGGCCCTGCTTGGCTAAGCTGGTGTGCCCCAGCTTGCCTGCGTTATAGCTTGTACCCATGTTTGGTGGTTCCCAAGCTCTTGTACTG
CACCCAGAAAGAATGAAGATATGCTGGACATGGAAGGGTGAGCAGGGTGGAGA**AGAC**TCTTATTGAGCGACGAAACAGCTTTCAGCAGAGAG
GGGACACGGGGGTGGT**CCCCCTACCC**AAAGGTGGGAAAGTCCCTCATGTGGCTGGGTCCGGGCCTTTTATGGACTCAGGATGGGGAGTGCA
TGCTGATTGGTTTGTGAGTATGCAAAAAAGGTTAAAGTGA**AGAC**ACCACTCAAAGGCGGGCATGACAATGCAGAAAACAAATTAGGAAAGGTT
AGGTATATGTCAAATAGGTGCAGGGTGGGAACAATCTGAGGAAAGTGTGCCAAACAGGA**AGAC**GAATTCTCCATCCGATCTGGGGATTTAACT
TGTAGTTTGGTGTTCAGGCTTTAAACT**GTCT**TCGGCTTGGAGGTGGGGCTTCACCAGGGACCTGCCCCTATTTGCCCAGGCATTTGGCTGCCT
CCTGCTGCTCTCAATAGTGAGCTAGGATCATGCCAGTGCACTCCAGCCTGGGCAACAGAGTG**AGAC**CCCATCTCAAAAAAAAAGAAAAAAAAG
AAAGCAGTTTCCTCAGGGGAGAGCCAGGTTTCGGGTACAGCAGGAGGGGAAGAGAATGGAAAAGAGGTTGAGGAAATGGTGAATTATGCTGA
TAATAAAACCAAGTTCCAGAAAGTTCCAGATGGCACAGTAGAAGGGTTTTTGGAGTTGGTGAAGAGTGAGAAGTGCCTACTCTGGCTGACTTA
AGCACAGAATTTGAGGGCAGGATATCAGTGAGGGCCCTTGGGAGCTTGGAGAACTAAGCCTGGAAAACAAGAGCAAGGTCATTGCCAAACTC
ATAGCACAGAACTGGACTTGTGA**AGAC**ACCACTGCTGCAGTTGGGCGCTGAGGGCCTCGGGGCCTGCACCGTCCCTGCCAGCCAGGGACCC
TGGCCGCTGCTGCTGCTGCCCTTGAAACTGCCGACCGACACCCTTCTCCTCTGGCCTGTTTCCTTCACCTGCTCCCACTGTAAGGGCTGGGTT
GGTGCACCTGAGTTGCTGAATGGAGCTCACAGGCCTTCACTCTCACTTCCAGGCAGGCTGGGGAGTGGGCACAGGGCATTTCCAAATTCTGC
AGTCAATGCGGTTCTTCCTTCTATCAACATTCATAAGGCCGGTATGCGTTATAGGGTTTTTATTACAAACTACAGAGCTTCCTCTCGGTCATATA
AGCAGAAAGGTTCTTATTATAGGACATTAACACACAGAATCTCAGGGAGGACAAGAGATGCAGCTTTGGAAACTATATAGCCAAGAACAGCACA
GTGACCGCAGGCCA**CCCCTTGGTG**CAATTTTAACAAAGCCACCACTCTG**CCCCAGCG**TGGGCACCAAGTACACTCAGGACC**AGAC**CAGAGAA
GCTCCATCACAGCCTCCCCAGAAAAACTAGATGCTGCTGCTTCTGCCTTCGCTGAAAGAAAGTTCCCGCTCACACAGCTGTGGCTTTGGGCTG
CCCATCCCCATTCCCAAATCCCTGGCGAGTATGCCCAATT**GGCAGA**GCCCGTGCACCTGGCCAGCATTAAGGAAGCCTGGGCAACATGTTGT
TTGTGGATTCTACTGTGAGAAGGTAGAACTCAAAACAGTAGTAGTTCTCAAACAGTGAGAACTATCAAAATAGAAGATGTTGAAAGGATGTTGT
GTATCTCTGAAGCAGAACTTCATCACTGCCAGATGGAATTTTCTCTTCTTCTCTACTCTACTCTTCTCTTCTCTTCTCTTCTCTTCTCTTCTCTTCT
CTTTTCTCTTCTCTTCTCTTCTCTTTTCTTTTCTTTCTTTTGACAGA**GTCT**TGCTCTGTTGCCCAGGCTGCGAGTGCAGTGGCATGATCTTGGCTT
ACTGCAACCTCTGCCTCCCGGGTTCAAGCAATTCTCCTGCTTCAGCTTCCCAAGTAGCTGGGATTACAGGTGTGTGCCACCACGCCCAGATAA
GTTTCGTATTTTTAATAG**AGAC**GGGGTTTCCCCATGTTGGCCAGGCTG**GTCT**CCAACTCTTAACCTCAGGTGATCCACCCACCTCAGCCTCCCA
AAGTGCTGTGATTACAGGTGTGAGCCACCATGCCCGGCTGGAATTTTCTGTATGTAAGAAAAGGTTATAGATGTTAGGAAGCTGGAAAATGAC
AAATAAGAAAAACGGCAAGTAGCCACCACCACTATTCCCAATCTCATTCTTCTCACTGCTCCAGAAGCCACCAGCCACTACCATTTGAATTCATT
ATTCCACTAAAATGGAAGATCCTTGACGGCCCCTATATTT**GTCT**GATTTATTTAACAATTGATCCCAAGTGCCTAGAACAGTGCCTGGCACATAG
AAGATGCTTGATACATATTTGCTGATGGAATGAA**AGAC**GCTCGTTCATTTGCCGCATTGAAAAAA**AGAC**AAAACTTCAAGTGGTTTGTAGGTTTA
GAAAACTGCCAGAGGTAGCTTTACTTTGAACTCCATGAGTATCTACAGTAGAGAGAAGCAGCAGTGTAAAGTCCTCCCTCCCAACCCCTAGTG
GTTAAACAGATTTTATAAAATAGGGTTTGACAATCAAGGACTAGCAGGAAGTTGCAGAAGGCACAGGAAT**GGCAGA**AAACCCAAAATAAACCTT
AATGAGGTCAGTGTACATTCATTATGAATTTAGTGATTGAAATCAGGTGTAATTTATAAATTTTCACTACTACATTAGCCATCTTTTATGATTCATG
CTATCACCTAAAACATATTTCATAAATATTAAAATTAGA**AGAC**ATAGGGGTTAGGATTTACATTTCAAAAAGCCACCCAGTTCCCATAGCCCTGCT
GGGTAAGCCCTTATCTCTCTGTACCTTAGTAGCAGAAAACACAGGAGAAGCACGACCATGAAAAAGGGCAAAGTGCAGTGAAATGCAATTG**AG**
ACCTTTAATCTTTGCCTAAGCAATTCAGCCAGAAAGCTCTGTAACTCACAAGGCATTATAAAAATATTCCACACCAGAGCTATTTACAAGTTAACT
GCTCCTGAATAAATAAAAGTCCATGTGATAGCTTCATGGCTTTCTCTTAACCAAAGAGGAAAGTAAAACTCATATTTCTATGTTTTTAAATTTTTCT
TCTCAAAAGGGGGCTTTATGAAATCTAGTTCTGTAGGTATGTCGTTCCTCTACACTTTACTGCTTGTATAAATATACTTACGTATTCATAAGCAAT
ACTGTGCTAGATGCAGAATTCAGATGCAGGCAACAGAAATCACTGTAGTTATTTTAAATAGAAACAGAAGAAAGGGATTTAAGATGAG**AGAC**TG
CAAGCCTACACAATCTTTGAACGGAATGGAGGAGTGAGCTCC**AGAC**TGAGCCTTTAGGAAGGACTCCCAGAAAACACCACCATAGAACTAGAA
GCTGCTATCTCTGCCTCAATCAGCAGGCCACCGCTAGAAACACGAACATATGGTTTGGCTGGGAGCCAAGGATCTGGAAGCAACTCCGTG**GT**
CTCGGGACTGAGCCACTGAGTCAATGGGGATGCCAGTTTGTAAGATAGGGAACACACTAGGAGGAACAGGCGTGGGAAGTAGAAATGCAGA
GAGAGTTCTGTTTTAGCCATGATAAATTTGAGATGCCTGTT**AGAC**ACTCAAGAGAAGATGTTCAGTAAGAAGTTGGATATTGACGTTGAGTAAG
ATGTTGAGTAAGAAGTTGGCAA**GTCT**GGAGCCCAAAGGAAACTCTAAGTTGGAGATTTAATTTCTTTCTTTTTTTTTTTTTGGAGGTAGA**GTCT**TG
CTGTGTCACCCAGGCTGGATGGAGTGCAGTGGCACAATCATGGCTCACTGCAGCCTCCACTTCCTGGGCTAAAGTGATCGTCCCACCTCAGC
CTCCTGAATAGCAG**AGAC**TACTGGCATGCACCACCATGCCTAGCTAATATTTTATTTTTATTTTTTATAGAGAGAAT**GTCT**TGCTTTGTTGCCCA
GGCTT**GTCT**CAAACTCCTGGCCTCAAGCAATTCTCCCACCTCAG**CCCCAAAGTGCTGGG**ATTACAGGTGTGTGCCACCATGCCCAATCTGGAG
ATTTAACAGGTTTCATACTATATAGGTCAGGCTGCAATTTTCTTTCTATTTTTTAAAATTCAATTATACCTTTGAGATCAACTAACTTCATGAGAAA
CTAGCTTGCTGTACTCCCTTCTCTGCCTATCTTTCA**GTCT**TCAAATCCAGAGTAATCCAGTTTCTACCATAAAGGGCCCACAAAAAGTGTTCTGA
AACATCCGGTGTCATCCAAGTCCAAAATTGGACAGCTTTTTTTTCAGTTCTTCCTGTAACATTTGGCAGTGTTGGCCACTCTCTGCTTCTTTGGC
AAGAATCTCAGAAAACACTAATAACTGCTCCAGGCAGGAGCCTGGAAGAGGTTACCAAGCTAGAGAAGGAAGCAACAGCATAGCTTGCTAAGG
GCAAACAGCCCAGAGGAAACCCACGGCTCTGGTCAATCCTGTTGTTGATTGCTAGATCCAG**AGAC**TGTTGT**AGAC**CTCCCAGTTACAGTGAG
GTATCTGACAA**CCCCTCACAAGG**TTCTTGAAGCTCCGTGGTGGAGGGTATCTTGGATAAATCCACAG**AGAC**ACATTCAAGAGTAAATTCTCCA
CTGTTGG**AGAC**CTCACTCACTCTGTTTG**GTCT**CCACCAA**AGAC**ACGGTTTTTAATTCATGGAGCTGCACTACAACAGCTCAAGAACTCCCAGCC
AAAGTTTTAGTTTTTCTAGGAACATAAACGAGCCAATAGTGTGGAAATCGGTGATTAAAAACAAACCTCGCACATTTTA**AGAC**AGAATTATTACA
AGTAGCATGTACAAAAGGAAAATAATAGCCTACATTTAGTTCTTCTAAGCTTCATTCCACAAGAGATTGAAATCGAAGTATTCTGGGCTGGCACA
CGATAGCTGGAACATCATATTTTATTCTAGAAACACATTTGCCTTGGCAAAGAAGAGCGTGCCTAGAGGAGTGGTCAGGATAGTGAGGGATCT
GTGATCCTTCGTTCTGAATCTAGAAAAGTCACTGGATATGCCCCTCCCCCGCCCCCCAACACG**GTCT**TATGTTCTGAATGTAGAAGTCACTGGA
-460-TGTGCTGACACACACACACACACACACACACACACACACACACACACACACACACACACACAGA**GTCT**GTGGCCTCTTTCCCAGGCATTCCAA
-368-GTCCAGCAAGTTCGCAGGGAGCTGTCAGTCCGTCCAGGAAGGCCCGGGCCTGGGTTTGCCTCTTCAAGCAGCTACTGCAGGGGCGTGGGG
-276-AGGGGGCATAAG**AGAC**TTTGGACTTTCCTTTG**AGAC**AGTAGAAAGCGTTACATCCAGAGGCGAGATTCTAGCCTGGGGT**CCCCGCCTTCCC**G
-184-GCCTCCTCTTCCTCTCCCTCTGACTCCCTTTCCTGTG**CCCCTCCCCC**TGCCTCTTTCCCGGCCAGAGTCCAGCCTTAACCCG**GGCAGAGG**GC
-92-GGAGTCCCGTTAAGGGGGTGTGGGGAGGAGGCGGGGCCAGGGCAGGGGCGG**GGCAGAGC**CGGGCCAAGCTGGGCGGGTCATGCGCCCT
-4-GGC**CTTCGCG**CATCTCCCAGGTTAGCTGCGTGTCCGGGTGCTAGGCTGC**AGAC**CCGCCGCCATGACGCTGCGCGCGGCC**GTCT**TCGACCTT
+89-GACGG

Supplementary Fig. 3. **The sequence of the human sEH promoter** (approximately 7 kb). Related to Figure 3. Smad binding elements (AGAC/GTCT) are highlighted in blue, SP1 binding element in orange, transcriptional start site (defined as +1) in red.

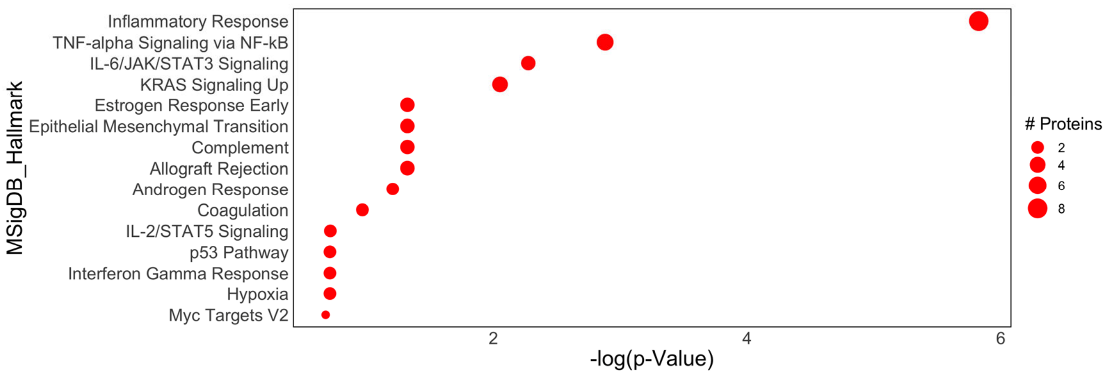

Supplementary Fig. 4. Analysis of gene expression in M2c macrophages from wild-type and sEH$^{-/-}$ mice. Related to Figure 4a-b. Hallmark gene set analysis (Molecular Signatures Database) for biological states and processes using the RNA-seq dataset.

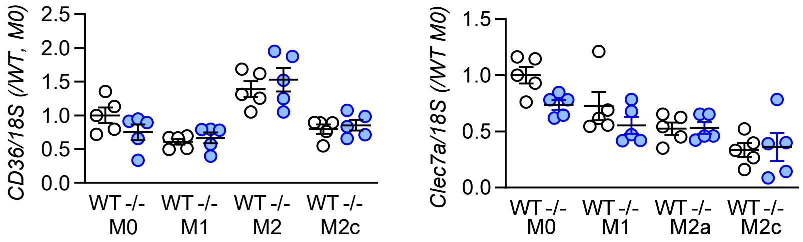

Supplementary Fig. 5. Phagocytosis associated receptor expression in macrophages from wild-type and sEH$^{-/-}$ mice. Related to Figure 4f-i. Expression of *Cd36* and *Dectin* in bone marrow-derived macrophages from wild-type (WT) and sEH$^{-/-}$ (-/-) mice under basal conditions (M0) and following M1, M2a and M2c polarization; n=5-6 mice per group. Two way ANOVA followed by Sidak's multiple comparisons test.

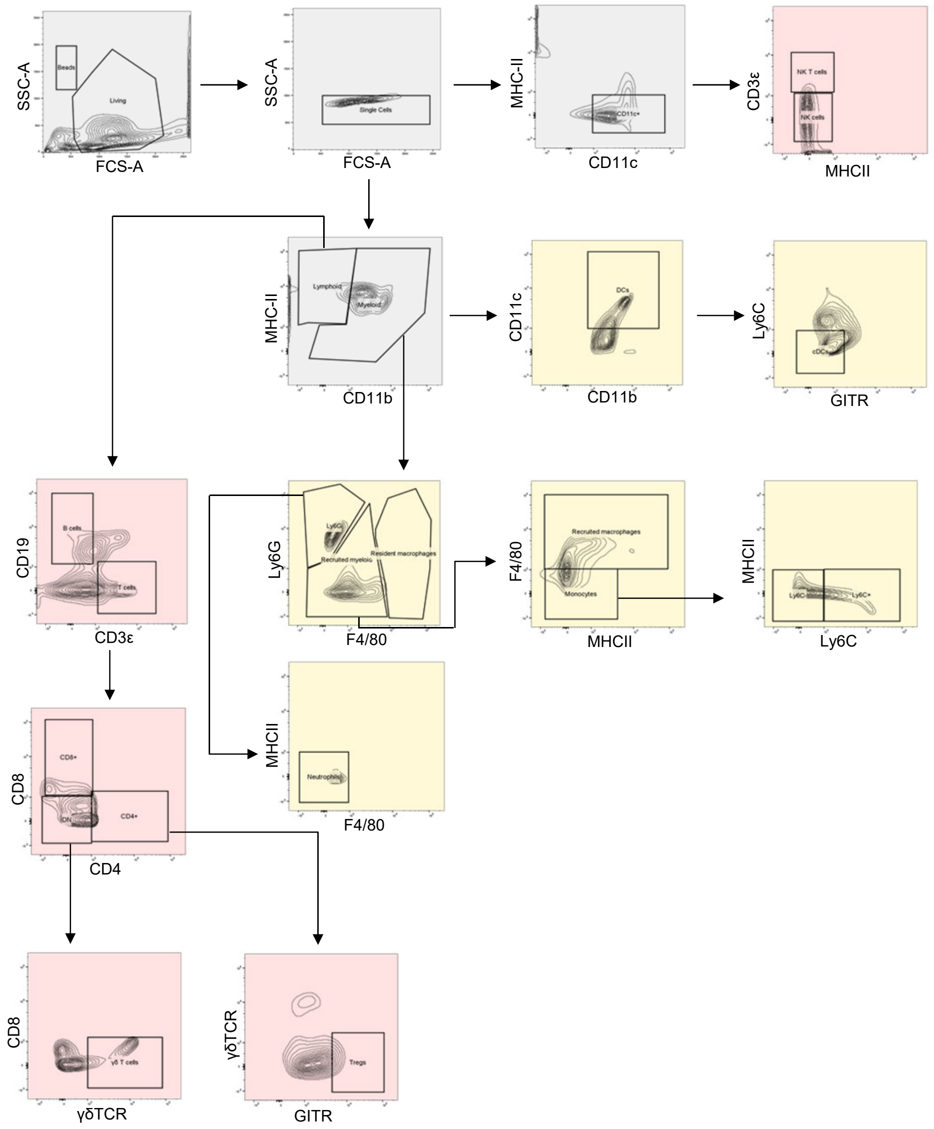

Supplementary Fig. 6. **Gating strategy of intraperitoneal cell populations**. Related to Figure 1. The example shown is from day 1. Peritoneal lavage obtained from the wild-type and sEH$^{-/-}$ was evaluated by flow cytometry. The analysis to evaluate immune populations was performed using the indicated gating strategy. Myeloid (yellow background) and lymphoid (red background) leucocytes categories were defined as the following subtypes: Neutrophils (CD11b+ Ly6G+), monocytes (CD11b+ F4/80low Ly6G- Ly6C+/-), macrophages (CD11b+ F4/80+Ly6G-), NK Cells (CD11c+ CD3ε+), NK T cells (CD11c+ CD3ε), dendritic cells (CD11c+ CD11b+ Ly6C-), T cells (CD11b-, CD3ε+), B cells (Ly6G+CD11b- CD3ε-).

Supplementary Table 1. PCR primers. Related to Figures 1 and 2.

Gene	Forward	Reverse
18S	ctttggtcgctcgctcctc	ctgaccgggttggttttgat
Ephx2	acgaccgtgctgagagagat	ttcagattagccccgatgtc
Nos2	gtggtgacaagcacatttgg	gttcgtcccccttctcctgtt
Tnf	ggccttcctaccttcagacc	ccggccttccaaataaatac
Il1b	caggcaggcagtatcactca	agctcatatgggtccgacag
Arg1	gtgaagaacccacggtctgt	ctggttgtcaggggagtgtt
Ym1	ctggaattggtgcccctacaa	tcataaccaacccactcattacc
Fizz1	cccttctcatctgcatctcc	cagtagcagtcatcccagca
Mrc1	tggatggatgggagcaaagt	gctgctgttatgtctctggc
Vctn1	aaagacgacctctcacagca	catactgaaggctccggtct
Snai1	tggctgatggagtgcctttgta	agccagtgggttggctttagtt
Id3	cctgcagcgtgtcatagactacat	agatcacaagttccggagtgag
Tlr2	ccctgtgccaccatttcc	ccacgcccacatcattctc
Tlr4	cagcaaagtccctgatgaca	agaggtggtgtaagccatgc
Cd36	gttgatactatgcccgcctct	gtttcccacactcctttctcc
Clec7a	tctcagccttgccttcctaa	catggcccttcactctgatt
Nlrp3	aaggcttgtgtgggaccaaa	aggaggggcaggagtaagag
